# The transcription factor ZEB2 drives formation of age-associated B cells

**DOI:** 10.1126/science.adf8531

**Published:** 2024-01-25

**Authors:** Dai Dai, Shuangshuang Gu, Xiaxia Han, Huihua Ding, Yang Jiang, Xiaoou Zhang, Chao Yao, Soonmin Hong, Jinsong Zhang, Yiwei Shen, Guojun Hou, Bo Qu, Haibo Zhou, Yuting Qin, Yuke He, Jianyang Ma, Zhihua Yin, Zhizhong Ye, Jie Qian, Qian Jiang, Lihua Wu, Qiang Guo, Sheng Chen, Chuanxin Huang, Leah C. Kottyan, Matthew T. Weirauch, Carola G. Vinuesa, Nan Shen

**Affiliations:** 1 Shanghai Institute of Rheumatology, Shanghai Renji Hospital, Shanghai Jiaotong University School of Medicine, Shanghai, China; 2 Centre for Personalised Immunology (CACPI), Renji Hospital, School of Medicine, Shanghai Jiao Tong University (SJTUSM), Shanghai, China; 3 Shenzhen Futian Hospital for Rheumatic Diseases, Shenzhen, China; 4 State Key Laboratory of Oncogenes and Related Genes, Shanghai Cancer Institute, Renji Hospital, Shanghai Jiaotong University School of Medicine, Shanghai, China; 5 Center of Autoimmune Genomics and Etiology, Division of Human Genetics, Cincinnati Children’s Hospital Medical Center, Cincinnati, Ohio, USA; 6 Department of Pediatrics, University of Cincinnati, Cincinnati, Ohio, USA; 7 Shanghai Institute of Nutrition and Health, University of Chinese Academy of Sciences, Chinese Academy of Sciences, Shanghai, China; 8 Francis Crick Institute, London, UK; 9 Shanghai Key Laboratory of Maternal and Fetal Medicine, Clinical and Translational Research Center of Shanghai First Maternity and Infant Hospital, Shanghai, China; 10 Frontier Science Center for Stem Cell Research, School of Life Sciences and Technology, Tongji University, Shanghai, China; 11 Department of Medical Genetics, Capital Institute of Pediatrics, Beijing, China; 12 Center for Immune-Related Diseases at Shanghai Institute of Immunology, Ruijin Hospital, Shanghai Jiao Tong University School of Medicine, Shanghai, China

## Abstract

Age-associated B cells (ABCs) accumulate during infection, aging, and autoimmunity, contributing to lupus pathogenesis. Here, we screen for transcription factors driving ABC formation and find ZEB2 is required for human and mouse ABC differentiation in-vitro. ABCs are reduced in *ZEB2* haploinsufficient individuals and in mice lacking *Zeb2* in B cells. In mice with TLR7-driven lupus, ZEB2 is essential for ABC formation and autoimmune pathology. ZEB2 binds to +20kb *Mef2b’s* intronic enhancer, repressing MEF2B-mediated germinal center B cell differentiation and promoting ABC formation. ZEB2 also targets genes important for ABC specification and function including *Itgax*. *Zeb2*-driven ABC differentiation requires JAK–STAT signaling, and treatment with JAK1/3 inhibitor reduces ABC accumulation in autoimmune mice and patients. Thus, ZEB2 emerges as a driver of B cell autoimmunity.

Age-associated B cells (ABCs) are a distinct effector B cell subset found at increased numbers in aged female mice, infection models, and systemic autoimmune diseases (1). ABCs are identified as CD11c^+^CD11b^+^CD21^−^CD23^−^T-bet^+^ in mice (2, 3) and CD11c^+^CD21^−^CD27^−^CXCR5^−^ FCRL5^+^IgD^−^T-bet^+^ in humans (4, 5). In autoimmune settings, these B cells are enriched for autoantibody specificities and are thought to be antigen-experienced. Moreover, there is evidence that ABCs can persist in tissues and rapidly differentiate into antibody-secreting cells (ASCs) upon antigen re-encounter or innate stimulation (1).

The transcription factors (TFs) T-bet, IRF5, and IRF8 are highly expressed in ABCs and have been put forward as functional regulators of ABC differentiation (6–9). However, except for IgG2a/c isotype switching, T-bet is dispensable for ABC accumulation and maintenance of ABC features (9–11), and IRF5 and IRF8 are broadly expressed in other B cell subsets and have been reported to be involved in cell activation, proliferation, differentiation, and function (12–14). To determine the TF(s) essential for ABC formation, we screened all TFs expressed by these cells and identified ZEB2 as the key regulator required for ABC specification and differentiation in mice and humans.

## Screen for TFs directing ABC differentiation

**Fig. 1:**
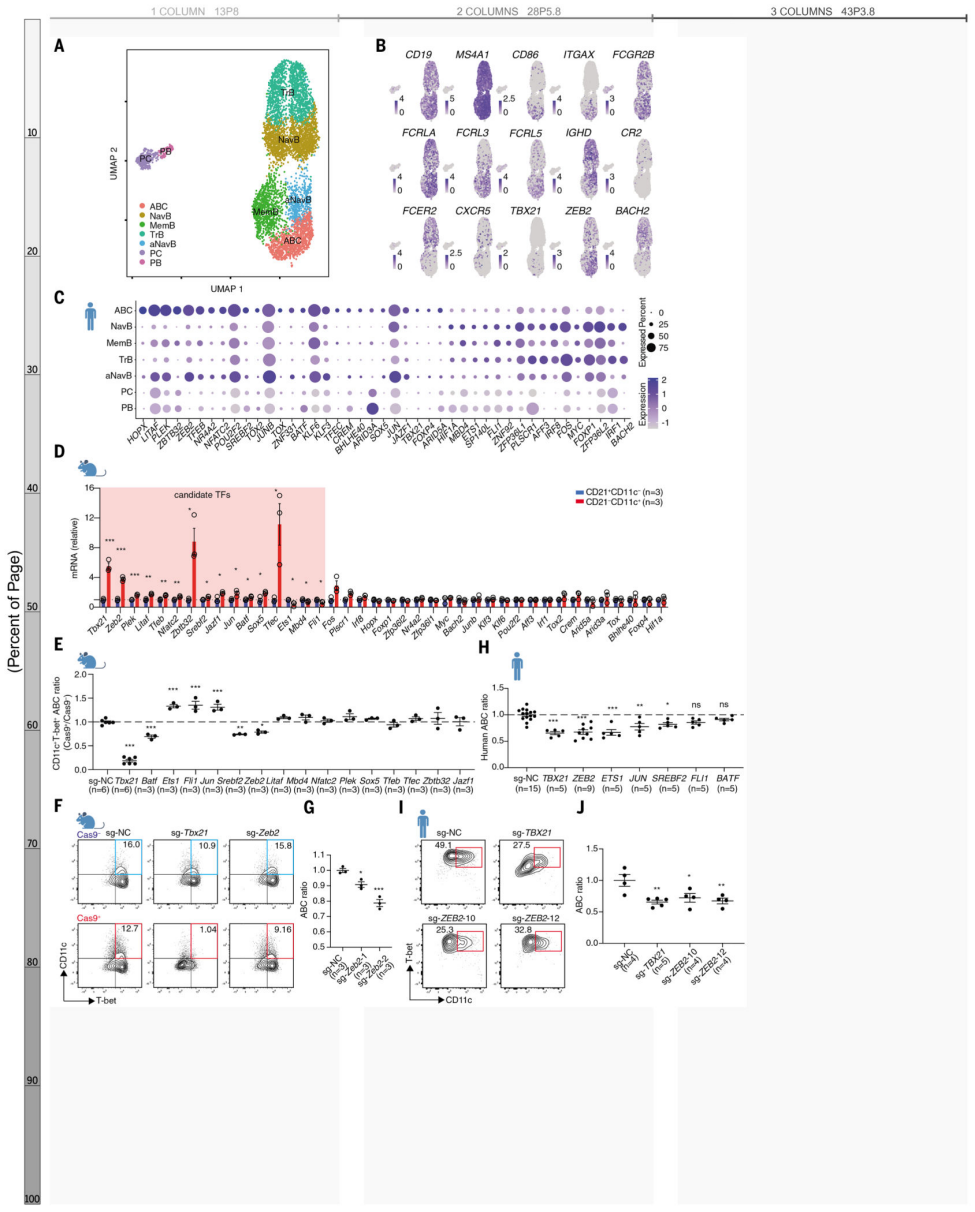
CRISPR/Cas9 based screen of transcription factors for ABC differentiation. (**A**) Single-cell RNAseq of CD19^+^ peripheral B cells isolated from a patient with new-onset SLE. Seven clusters were defined as transitional B cells (TrB), naïve B cells (NavB), activated naïve B cells (aNavB), age-associated B cells (ABC), memory B cells (MemB), plasmablasts (PB), and plasma cells (PC). (**B**) UMAP plots of select genes expression distinguishing ABCs. (**C**) Dot plots of 43 differentially expressed transcription factors (TFs) in ABCs. (**D**) Relative expression of mouse homologue genes (encoding equivalent TFs in (C)) in CD19^+^CD11c^+^CD21^−^ and CD19^+^CD11c^−^CD21^+^ B cells sorted from bm12-induced lupus mice. (**E**) Mouse ABC ratio in groups targeting indicated genes. The ratio defined by comparing Cas9^+^CD11c^+^T-bet^+^ to Cas9^−^CD11c^+^T-bet^+^ in the coculture was normalized with sg-NC. (**F**) Flow cytometry plots of ABCs (CD11c^+^T-bet^+^) derived from B cells transuded with sgRNA targeting *Tbx21* or *Zeb2*. (**G**) ABC ratio in groups with two distinct sgRNAs targeting *Zeb2*. (**H**) Human ABC ration in groups targeting indicated genes. The human ABC ratio was defined by normalizing the frequency of CD27^−^IgD^−^CD11c^+^T-bet^+^ B cell with sg-NC group. (**I** and **J**) Representative plots (I) and ABC ratio (J) from B cells electroporated with Cas9-gRNA (RNP) complex targeting *TBX21* and *ZEB2*. n represents distinct samples (biological repeats). Data are representative of 3-4 independent experiments. Data are mean ± SEM values. *P<0.05, **P<0.01, ***P<0.001, ns, not significant, unpaired Student’s *t* test (D) and ordinary one-way ANOVA with Dunnett’s multiple comparisons testing (E, H, G, and J).

To gain insights into the nature of ABCs, we sorted peripheral B cells from a patient with new-onset SLE (table S1) and performed droplet-based scRNA-seq. Seven distinct clusters were revealed by unsupervised clustering with a two-dimensional uniform manifold approximation and projection (UMAP) (Fig. 1A and fig. S1A and B). These clusters were assigned to known peripheral B cell subsets including transitional B cells, naïve B cells, activated naïve B cells, ABCs, memory B cells, plasmablasts, and plasma cells by comparing differentially expressed genes with established landmark genes (5, 15) (fig. S1A and C). ABCs preferentially expressed genes encoding key surface markers *CD19*, *CD86*, *FCRLA, FCRL3/5, FCGR2B, MS4A1* and *ITGAX,* and they lacked *CD27, CR2, CXCR5,* *FCER2*, and *IGHD* (Fig. 1B and fig. S1C). We found 43 differentially expressed TFs: 27 upregulated and 16 downregulated (Fig. 1C). We sorted murine CD19^+^CD11c^+^CD21^−^ B cells from the bm12 induced lupus mouse model and validated 40 murine homologues of the differentially expressed TFs identified in the human scRNA-seq data (Fig. 1D, and fig. S2A-C). Among these, 13 upregulated (*Tbx21*, *Zeb2*, *Plek*, *Litaf*, *Tfeb*, *Nfatc2*, *Zbtb32*, *Srebf2*, *Jazf1*, *Jun*, *Sox5*, *Tfec*, and *Batf*) and three downregulated TFs (*Ets1, Mbd4,* and *Fli1*) were identically regulated in human and mouse ABCs and were therefore considered potential transcriptional regulators of ABC differentiation.

To identify which of these 16 TFs were driving ABC differentiation, B cells from Cas9 transgenic mice and CD45.1 congenic mice were retrovirally infected with sgRNA plasmids targeting each TF and co-expressing blue fluorescent protein (BFP), and cultured with the ABC differentiation cocktail (7, 16) (fig. S3A). The ratio between live BFP^+^ CD45.1^−^ and BFP^+^ CD45.1^+^ ABCs was determined and genome-editing was validated with a sequence specific for *Itgax* (fig. S3B and C). We identified seven TFs that could significantly (*P*<0.05) alter ABC formation (Fig. 1E and F, and fig. S3D). Except for *Zeb2*, ablation of the other six TFs predominantly influenced cell viability (fig. S3E and F). Two different sgRNAs targeting *Zeb2* in separate Cas9^+^ B cell cultures led to reduced ABC formation (Fig. 1G), excluding an off-target editing effect. To determine whether any of the other six TFs was required for human ABC lineage formation, we transduced Cas9-guide RNA ribonucleoprotein (RNP) complexes by electroporation and cultured edited cells with the ABC differentiation cocktail (4–6). (fig. S4A and B). Ablation of *TBX21*, *ZEB2*, and *SREBF2* dampened ABC differentiation in both human and mouse B cells but *TBX21* and *SREBF2* deficiency also led to altered B cell viability (Fig. 1H, and fig. S4C-E). Gene editing of *ETS1* and *JUN* had opposite effects and *BATF* and *FLI1* did not change human ABCs (*P*<0.05) (Fig. 1H and fig. S4C). Two different guide RNAs targeting *ZEB2* impaired ABC induction (Fig. 1I and J) emerging thus as the most promising putative ABC transcriptional regulator.

## *ZEB2* happloinsufficiency impairs human ABC formation

ZEB2, a member of the zinc-finger E homeobox-binding protein family, is pivotal in early fetal development and cancer progression by driving the epithelial-to-mesenchymal transition (17). Loss-of-function heterozygous gene variants lead to Mowat–Wilson syndrome (MWS), a rare genetic disorder in which *ZEB2* happloinsufficency causes intellectual disability, distinctive facial features, seizures, and a predisposition to Hirschsprung disease (18, 19). Though mouse studies have shown ZEB2's role in immune cell differentiation and function (20), insights into the immunological consequences of reduced ZEB2 function in MWS patients are limited.

**Fig. 2:**
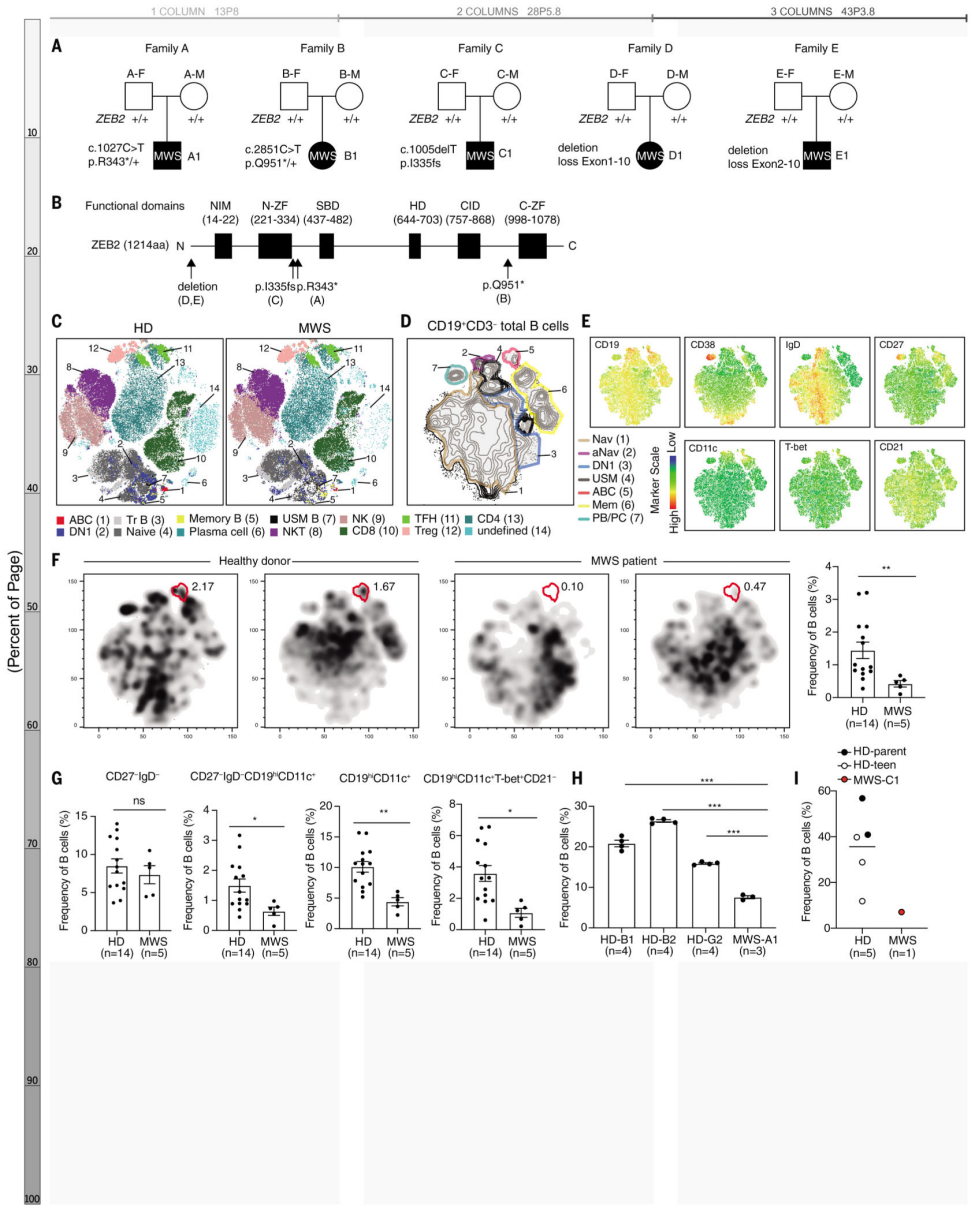
ZEB2 is required for human ABC formation. (**A**) Family pedigrees showing de novo heterozygous mutations of ZEB2 in Mowat–Wilson syndrome (MWS) patients. (**B**) Schematic of the general, linear structure of the functional domain composition of ZEB2 protein. The black arrows show the location of ZEB2 mutation described in (A). (**C**) t-SNE plots of lymphocytes clusters in PBMC of a healthy donor (HD) and a MWS patient by flow cytometry. (**D**) t-SNE plots of peripheral B cell clusters for MWS patients and healthy donors. Seven B cell clusters were identified based on lineage marker expression as naïve B cells (Nav), activated naïve B cells (aNav), CXCR5^+^ double-negative B cells (DN1), unswitched memory B cells (USM), age-associated B cells (ABC), memory B cells (Mem), plasmablasts (PB), and plasma cells (PC). (**E**) t-SNE plots of peripheral B cells displaying CD11c, CD19, CD21, CD27, CD38, IgD, and T-bet expression. (**F**) Representative t-SNE plots and frequency of ABCs (red frame) in peripheral B cells from MWS patient and HDs. (**G**) Frequency of DN, DN2, CD11c^+^B, and ABCs in PBMCs from MWS patient and HDs analyzed by manual gating. (**H** and **I**) Representative plots and frequency of in vitro–induced ABCs derived from B cells of MWS-A1 (H), MWS-C1 (I), and healthy donors. n represents distinct samples (biological repeats except (H)). Data are mean ± SEM values. *P<0.05, **P<0.01, ***P<0.001, ns, not significant, unpaired Student’s *t* test (G and H) with Welch’s correction (F).

We examined peripheral blood mononuclear cells from five unrelated MWS patients (tables S2 and S3) and identified 5 de novo heterozygous germline *ZEB2* variants through whole-exome sequencing (Fig. 2A and B). *ZEB2* deficiency dramatically decreased ABC frequency (Fig. 2C and fig. S5A). Detailed B cell profiling revealed seven prominent B cell clusters (Fig. 2, D and E). In MWS patients, ABC frequency was notably reduced by 71% accompanied by a significant decline in activated naïve B cells and ABC progenitors (5) (Fig. 2F and fig. S5B). Although switched memory B cells were decreased by 46%, DN1 B cells—an alternative trajectory for effector B cells—were increased (fig. S5B) (5). These changes were confirmed using manual gating with specific markers (Fig. 2G and fig. S5, C and D). We further studied ZEB2's regulatory effects on ABC formation by isolating B cells from MWS patients and inducing ABC differentiation in vitro. Corroborating our in vivo findings, in vitro ABC formation was also impaired (Fig. 2, H and I, and fig. S5, E and F). Thus, *ZEB2* loss-of-function variants confirm that ZEB2 is required for human ABC formation.

## ZEB2 determines ABCs pathogenicity in lupus

**Fig. 3:**
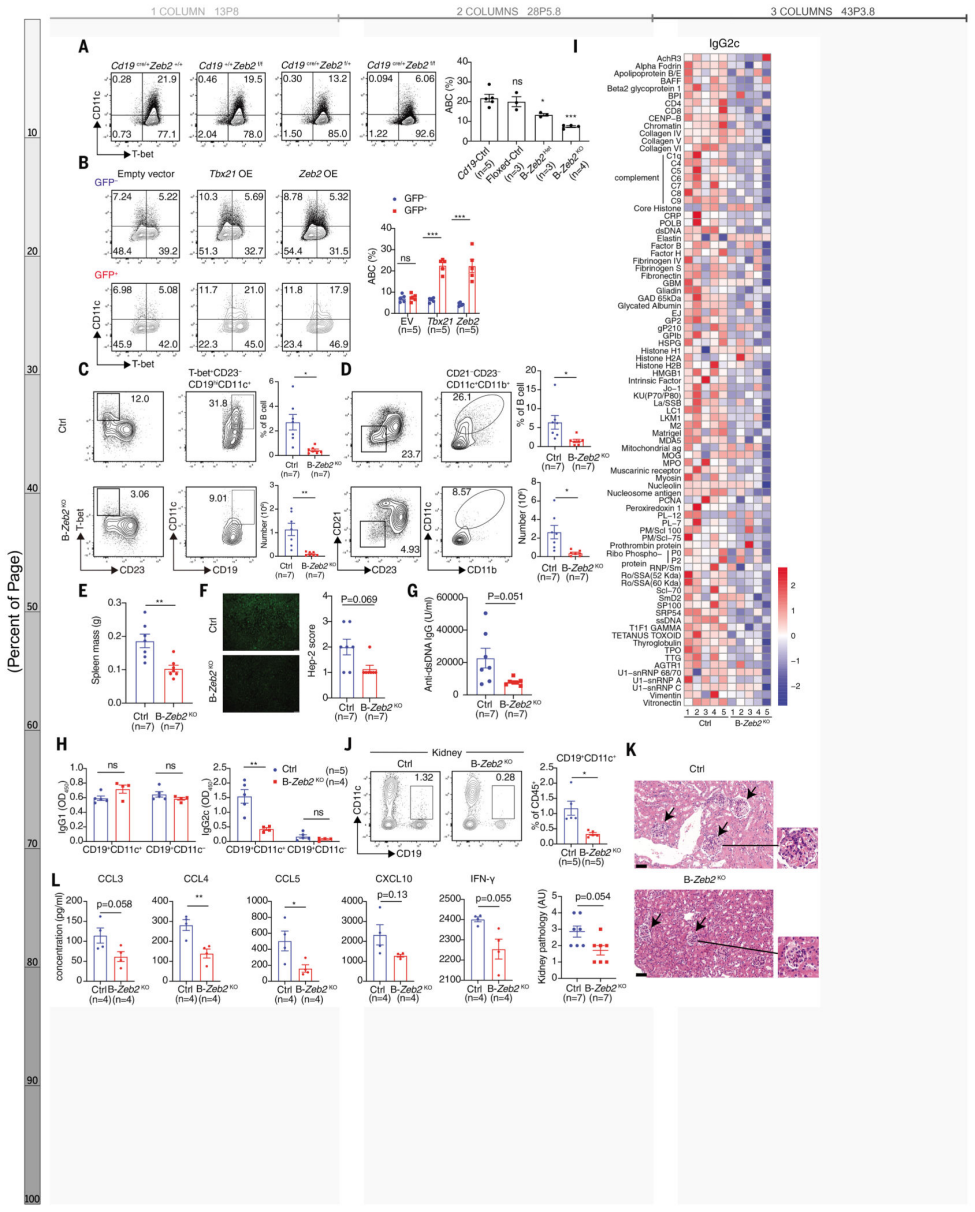
Zeb2 deficiency impairs ABC formation and alleviates lupus pathogenesis. (**A**) Representative plots and frequency of in vitro induced ABCs (CD19^+^CD11c^+^T-bet^+^) derived from splenic B cells of *Zeb2*^+/+^*Cd19*^Cre/+^ (CD19-Ctrl), *Zeb2*^f/f^*Cd19*^+/+^ (Floxed-Ctrl), *Zeb2*^f/+^*Cd19*^cre/+^(B-*Zeb2*^Het^), *Zeb2*^f/f^*Cd19*^cre/+^(B-*Zeb2*^KO^) mice. (**B**) Representative plots and frequency of ABCs (CD19^+^CD11c^+^T-bet^+^) in GFP^+^ (infected) and GFP^−^ (uninfected) B cells transduced with empty plasmid, *Tbx21* or *Zeb2* cDNA sequence. (**C** and **D**) Representative plots, frequency and absolute number of splenic ABCs identified by CD19^+^CD23^−^CD11c^+^T-bet^+^ (C) and CD19^+^CD21^−^CD23^−^CD11c^+^CD11b^+^ (D) in IMQ-induced B-*Zeb2*^KO^ and *Cd19*^Cre/+^ (Ctrl) mice. (**E** to **G**) Spleen weight (E), ANA (F), and anti-dsDNA (G) in serum from mice described in (C and D). (**H**) The IgG1 and IgG2c antibody titers in the culture supernatants from CD19^+^CD21^−^CD11c^+^ and CD19^+^CD21^+^CD11c^−^ B cells sorted from mice described in (C and D). (**I**) Autoantigen microarray showing the relative IgG2c-isotype autoantibody levels in the serum of mice described in (C and D). (**J**) Representative plots and frequency of renal ABCs (CD19^+^CD11c^+^) from mice described in (C and D). (**K**) H&E staining (right) and pathology assessment (left) of kidneys from mice described in (C and D). (**L**) The concentration of cytokine and chemokine in the culture supernatants described in (H). n represents distinct samples (biological repeats). Data are representative of 2-3 independent experiments. Data are mean ± SEM values. *P<0.05, **P<0.01, ***P<0.001, ns, not significant, unpaired Student’s *t* test (B, E, H, L for CCL3, CCL4 and CCL5) with Welch’s correction (C, D, G, J, and L for CXCL10 and IFN-γ), Mann–Whitney *U* test (F and K) and ordinary one-way ANOVA with Dunnett’s multiple comparisons testing (A).

To further explore ZEB2’s role in ABC formation, we generated mice selectively lacking *Zeb2* in B cells by crossing *Zeb2* floxed mice with *Cd19*-cre mice (fig. S6, A to C). *Zeb2* deficiency in B cells reduced ABC differentiation by over 50% (Fig. 3A). Even mice hemizygous for *Zeb2* in B cells (B-Zeb2^Het^) displayed reduced ABC formation, providing evidence of *Zeb2* haploinsufficiency in mice. Moreover, overexpression of *Zeb2* in splenic B cells promoted ABC formation, indicating that *Zeb2* is sufficient for ABC differentiation in vitro (Fig. 3B).

ABCs are a unique effector B cell subset that arises during immune responses to nuclear acid–related antigens (1), developing separately from the germinal center (GC) pathway (21, 22). To investigate how ZEB2 impacts pathogenic ABCs, we investigated the consequences of *Zeb2* deficiency in B cells using two lupus mouse models (lupus induced by the TLR7 agonist imiquimod (IMQ) and bm12 cell transfer) as well as an acute LCMV infection model. B cell–intrinsic *Zeb2* deficiency significantly impaired ABC formation in all three models (Fig. 3, C and D, and figs. S6, D to F, and S7, A to C). Furthermore, GC B cells increased in B cell–intrinsic Zeb2-deficient mice in the acute bm12-induced and LCMV infection models (fig. S7 D and E) suggesting a competitive relationship between GC B cells and ABCs. *Zeb2* neither directly instructed GC B cell differentiation nor promoted antibody responses to an ABC-irrelevant protein antigens however (fig. S7F and G). Detailed profiling of ABCs in IMQ-induced lupus confirmed that ABCs were phenotypically distinct from CD38^−^GL-7^+^ GC B cells (fig. S8A). A significant proportion of CD19^hi^CD11c^+^IgD^−^ ABCs exhibited CD38^+^GL-7^−^ memory markers, while also displaying a unique hyperactivated state (CD95^+^CD80^+^) in comparison to other memory B cells (Fig. S8A). *Zeb2* deficiency selectively impacted the distribution and hyperactivation of CD11c^+^ memory-like B cells, without affecting the frequency of the CD11c^−^ memory B cell subset (fig. S8B). A subpopulation of ABCs acquired a phenotype (CD38^+^GL-7^+^) consistent with precursors of GC (pre-GC) B cells (fig. S8C), suggesting that like conventional memory B cells, ABCs could contribute to secondary GCs, seeding a chronic GC response. Notably, ABCs reside at the pre-plasma cell stage and can quickly differentiate into plasma cells (1). Such chronic GC responses and terminally differentiated plasma cells were reduced in IMQ-induced B-*Zeb2*^KO^ mice, likely due to reduced replenishment from ABCs (fig. S8 D to E). Thus, rather than broadly promoting all effector B cell responses, ZEB2 selectively drives ABCs and their progeny. Moreover, although these cells develop extrafollicularly, their progeny may participate in GC responses in the context of chronic inflammation.

TLR7-driven lupus is GC-independent and mostly ABC-dominant (21). We therefore examined whether ZEB2 regulated ABC-mediated autoimmunity in lupus induced by IMQ, a TLR7 agonist. *Zeb2* deficiency in B cells significantly ameliorated splenomegaly (Fig. 3E) and decreased serum antinuclear (ANA) and dsDNA autoantibodies (Fig. 3, F and G). ABCs are particularly pathogenic due to secretion of antibodies of the IgG2a/c isotype (1). Compared to non-ABCs, ABCs secreted the highest levels of IgG2c isotype antibodies upon restimulation. By contrast, residual ABC-like cells isolated from B-*Zeb2*^KO^ mice were unable to produce comparable amounts of IgG2c antibodies (Fig. 3H). Similarly, B-*Zeb2*^KO^ mice treated with IMQ produced much lower IgG2c autoantibodies (Fig. 3I). In lupus nephritis (LN), ABCs correlate with tissue damage (21, 23) and are known to produce proinflammatory cytokines and chemokines like CCL5, CXCL10, IFNγ, and IL-6 (8). In IMQ-treated B-*Zeb2*^KO^ mice, kidney-infiltrating ABCs were substantially decreased (Fig. 3J and fig. S8G) as was tissue damage (Fig. 3K). The residual CD11c^+^CD21^−^ B cells from B-*Zeb2*^KO^ mice also produced reduced quantities of CCL5 and CXCL10 ex vivo (Fig. 3L). Thus, ZEB2 is essential for ABC-mediated autoimmunity and the proinflammatory properties of ABCs.

## ZEB2 controls the lineage specification and cellular identity of ABCs

**Fig. 4:**
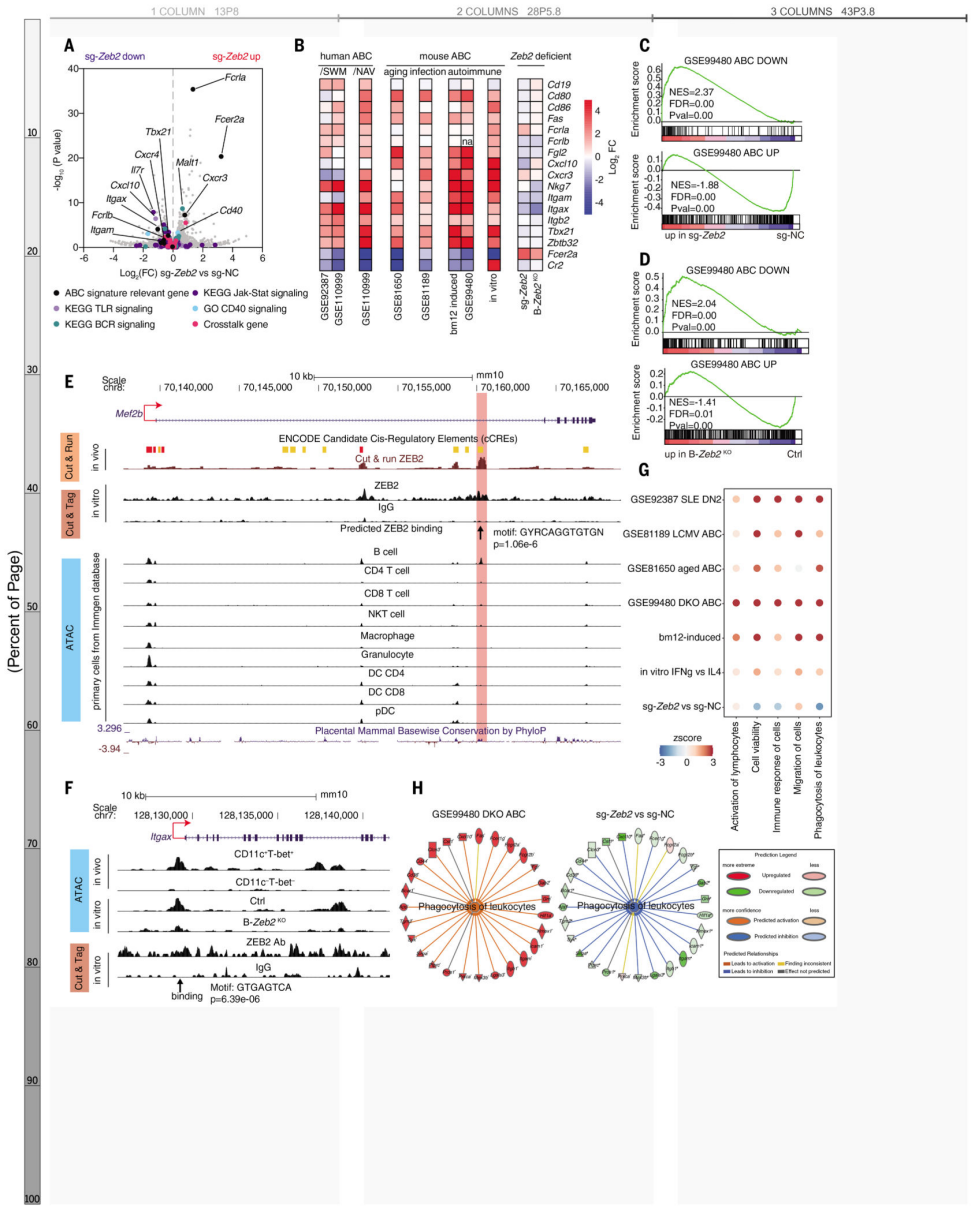
Zeb2 regulates specification and cellular identity of ABCs. (**A**) Volcano graph showing the transcriptional profiles in *Zeb2* edited B cells. ABC-signature genes, genes associated with BCR, TLR, JAK–STAT, and CD40 signaling, and crosstalk genes were labeled with colored dots. (**B**) Heatmap showing expression of representative ABC signature genes in public RNA-seq datasets: GSE92387, GSE110999, GSE81650, GSE81189, GSE99480 and RNA-seq of ABCs sorted from bm12-induced lupus mice (bm12-induced), ABC-polarized B cells (in vitro), ABC-polarized B cells derived from *Zeb2* edited B cells (sg-*Zeb2*) and *Zeb2*-knockout B cells (B-*Zeb2*^KO^). (**C** and **D**) GSEA showing the enrichment of the ABC-down geneset and ABC-up geneset from GSE99480 in ABC-polarized B cells derived from *Zeb2* deficiency B cells. *Zeb2* deficiency was mediated by sg-*Zeb2* editing (C) or knockout (D). (**E**) CUT & RUN, CUT & Tag, and ATAC-seq tracks display ZEB2 binding around the *Mef2b* locus, visualized by the UCSC genome browser. The chromatin accessibility in mouse primary immune cell subsets was from public Immgene database. (**F**) ATAC and CUT& Tag tracks display ZEB2 binding around the *Itgax* locus in ex vivo sorted ABCs and ABC-polarized B cells. (**G**) Dot plot showing the activation Z-score of predicated biological function in RNA-seq datasets mainly described as in (B). (**H**) Network diagram representing phagocytosis pathway in sg-*Zeb2* versus sg-NC ABCs by IPA. The color of each node indicates change in the expression: red (upregulated) and green (downregulated). The edges indicate the predicted relationship between nodes and biological function: orange representing activation, blue representing inhibition, and gray representing effect not predicted.

To investigate the consequences of ZEB2 regulation of gene transcription, RNA sequencing was performed on sorted *Zeb2* deficient B cells after in vitro ABC induction. ABC signature genes including *Itgax*, *Itgam*, *Itgb2*, *Nkg7*, *Tbx21*, *Zbtb32* and *Fcer2a/Fcer2* (encoding CD23a/CD23) (4, 5, 8, 24–26) were reversly expressed after *Zeb2* deficiency (Fig. 4, A and B). Gene set enrichment analysis (GSEA) revealed that *Zeb2*-deficient B cells lacked expression of the “ABC upregulated” gene set while it was enriched in the “ABC downregulated” gene set from the public dataset GSE99480 (8) (Fig. 4C and D).

To elucidate ZEB2's direct targets in ABCs, we performed high-throughput sequencing of the regulome by ATAC-seq, CUT & Tag, and CUT & RUN, leading to the identification of 4338 genes annotated by 6733 accessible sites with ZEB2 binding. Among the genes differentially expressed in *Zeb2*-deficient cells, we found 33 candidate direct targets: 22 repressed and 11 activated by ZEB2 (fig. S9A). Notably, *Mef2b*, an essential TF for GC development (27), was repressed by ZEB2. This direct regulation was mapped to a conserved region ~20 kb downstream of *Mef2b*'s exon 1 TSS, enriched with enhancer-associated features in both human and mouse (Fig. 4E and fig. S9, B to D). We validated ZEB2 suppression of *Mef2b* expression in *Zeb2*-deficient and *Zeb2*-overxpressing B cells and confirmed opposing expression patterns of *Zeb2* and *Mef2b* in ABCs and GC B cells from public datasets (22) (fig. S9, E to H). MEF2B can directly regulate *S1pr2* (27) and *Zeb2*-deficient B cells upregulated *S1pr2* expression (fig. S9E). Thus, ZEB2 appears to foster ABC differentiation by directly repressing *Mef2b* to constrain GC B cells, in alignment with our observations in the bm12 and LCMV models (fig. S7, D and E).

Additionally, ZEB2-specific peaks from CUT & Tag were matched to motifs of GATA3, FOSL2, and ZEB2 (fig. S10, A to C), consistent with existing ZEB2 Chip-seq data (fig. S10D). We identified a ZEB2-specific peak residing in the promoter of *Itgax*, containing a ZEB2-binding sequence (Fig. 4F). *Zeb2* deficiency altered the chromatin accessibility of the ABC-specific opening in the *Itgax* promoter, further confirming that ZEB2 controls transcription of ABC signature gene. CD11c (*Itgax*), an important alpha-subunit member of beta2 integrins, can pair with the beta-subunit CD18 to form heterodimeric cell surface receptors important for immune cell adhesion and recruitment to tissues (28), which is a unique property of ABCs (4, 21, 23). In kidney biopsies from patients with LN (table S4), CD11c^+^ B cells were found in affected tissues, constituting approximately 50% of total B cells with IgD^−^CD27^−^CD11c^+^ ABCs comprising about 20% (fig. S11, A and B). We validated the enhanced migratory capacity of ABCs in vitro, which was modulated by CD11c blockade and Zeb2 deficiency (fig. S11, C to G). Thus, Zeb2 plays a crucial role in orchestrating ABC specification by directly suppressing other effector B cell subsets and inducing the ABC signature.

## ZEB2 drives distinct functional properties of ABCs

To better define the function of ABCs, we applied ingenuity pathway analysis (IPA) to a public dataset (GSE99480) (8). Among the top 35 significantly increased predicted functions (table S5), ABCs shared five features: “enhanced viability”, “migration”, “activation”, “immune response”, and “phagocytosis/engulfment” (fig. S12A). These were validated accross several transcriptomes (Fig. 4G). Selected transcripts linked to these biological functions formed a network (fig. S12A). Pathway analysis further supported our finding that ABC-enriched pathways were linked to these five functional features (fig. S12B). ABCs have also been characterized by a hyperactivation state, long-term survival, and unique migration/distribution pattern in published studies (4, 5, 29). ABCs also exhibited a unique phagocytic capacity, identified by enriched phagosome formation and Fc-receptor pathways (fig. S12B). The ability of ABCs to both perform typical B cell functions and co-opt myeloid markers like CD11c, as well as cytotoxic molecules like NKG7, granzyme A, and perforin have been previously described (3, 4). In line with ZEB2’s critical role in ABC function, *Zeb2* editing in B cells dampened their viability, immune response, and phagocytosis/engulfment (Fig. 4, G and H).

To experimentally test the phagocytic capacity of ABCs, we incubated splenic B cells with apoptotic thymocytes labeled with pHrodo and monitored apoptotic cell internalization (fig. S12C). CD19^hi^CD11c^+^ B cells exhibited markedly enhanced uptake evidenced by both an increased pHrodo^+^ fraction and signal intensity (fig. S12D). In vitro–generated ABCs were also able to engulf apoptotic cells, which was dampened by *Zeb2* deficiency (fig. S12E and F). Thus ABCs exhibit unique biological functions that are regulated by ZEB2.

## The ZEB2–JAK–STAT axis governs ABC differentiation

**Fig. 5:**
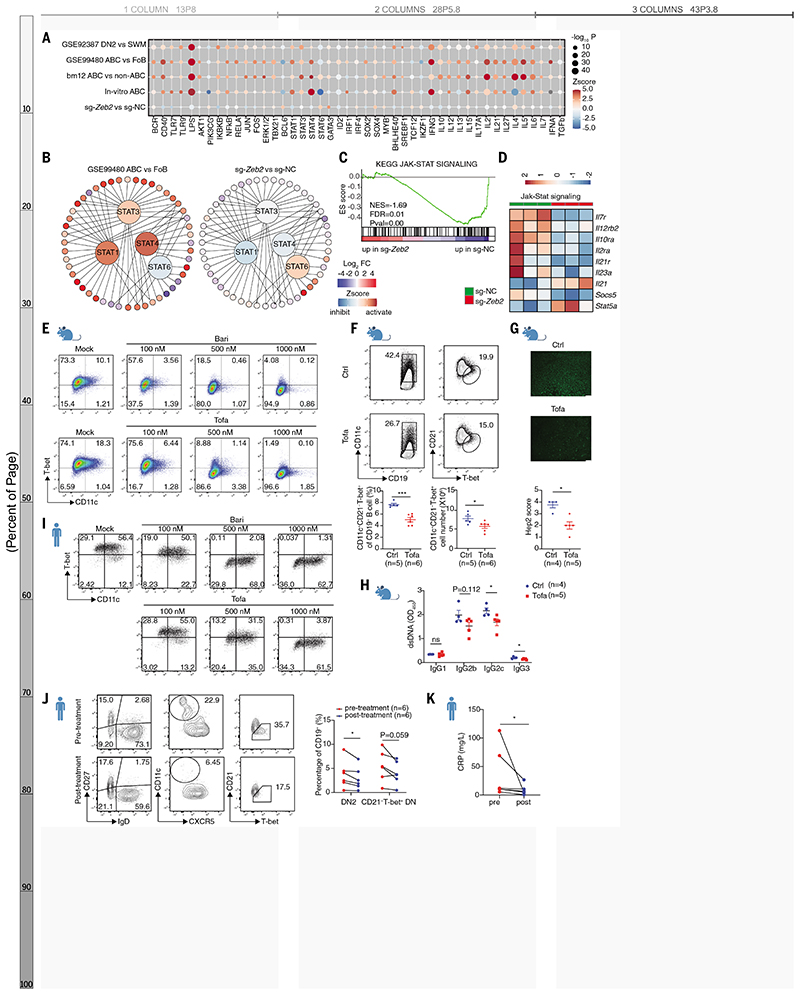
Zeb2-JAK–STAT axis controls ABC differentiation. (**A**) Activation *Z*-score heatmap for IPA-predicted upstream regulators in indicated datasets mainly described in Fig. 4B. *P*-values were log-normalized and presented by the size of the plot. The color of the plot indicates the activated (orange) or inhibited (blue) regulation of the predicted regulators. (**B**) Upstream regulator analysis showing STAT1, STAT3, and STAT4 activated and STAT6 inhibited in ABCs (GSE99480), whereas the opposite effects were observed in sg-*Zeb2* vs sg-NC dataset. The color of the surrounding circles indicates the change in the expression: red (upregulated) and blue (downregulated). The color of the center circles indicates predicted regulators: orange (activated) and blue (inhibited). (**C**) GSEA showing impaired JAK–STAT pathway in *Zeb2* edited B cells. (**D**) Heatmap showing expression of selected JAK–STAT signaling genes in sg-NC^+^ and sg-*Zeb2*^+^ B cells. (**E**) Flow cytometry plots of in vitro induced mouse ABCs with different concentrations of baricitinib (Bari) or tofacitinib (Tofa). (**F** to **H**) Frequency and absolute number of ABCs (F), ANA titer (G), and anti-dsDNA Ig titers (H) in serum from bm12-induced lupus mice treated with tofacitinib. (**I**) Flow cytometry plots of in vitro induced human ABCs with different concentrations of baricitinib or tofacitinib. (**J**) Flow cytometry plots of human ABCs in PBMCs from patients with rheumatoid arthritis before and after JAK–STAT inhibitor tofacitinib treatment for 4 weeks. (**K**) The change of CRP level in RA patients described in (**J**). n represents distinct samples (biological repeats). Data are representative of 2-3 independent experiments (E to I). Data are mean ± SEM values. *P<0.05, **P<0.01, ***P<0.001, ns, not significant, unpaired Student’s *t* test (F and H), Mann–Whitney *U* test (G), paired Student’s *t* test (J and K).

To elucidate the signaling pathways by which ZEB2 influences ABC formation, we performed upstream regulator analysis (URA) in IPA on both public and our own datasets. As anticipated, BCR, CD40, TLR and key downstream pathways like NF-κB and AKT were predicted to be activated in ABCs (Fig. 5A). Regulatory effects of cytokines IFNγ, IL-10, and IL-21, along with their JAK–STAT signals were also detected (Fig. 5A and B). Specifically STAT1, STAT3, and STAT4 were activated, whereas STAT6 was inhibited (Fig. 5, A and B) consistent with previous findings (1). *Zeb2* deficiency altered STAT signals (Fig. 5, A and B), reflecting opposite expression pattern of STATs’ target genes in *Zeb2* edited B cells (Fig. 5B). GSEA and KEGG analysis produced similar findings, supporting an important role of JAK–STAT signaling in ZEB2’s function (Fig. 5, C and D, and fig. S13A). We also confirmed that gene expression altered by *Zeb2* deficiency largely overlapped with the transcriptional program affected by inhibition of JAK–STAT signaling (Fig. S13, B and C).

JAK–STAT inhibitors, such as baricitinib and tofacitinib, have proven to be effective in dampening intracellular cytokine signaling (30, 31). We therefore tested their effects on mouse ABC formation and found they impaired in vitro ABC differentiation in a dose-dependent manner (Fig. 5E and fig. S13, D to G). Tofacitinib administration reduced ABC accumulation and splenomegaly, lowered autoantibody titers, and decreased ABC-relevant cytokines in a manner likely to be B cell–intrinsic (Fig. 5, F to H, and fig. S14, A to E). Human ABC differentiation was also inhibited by these drugs (Fig 5I and fig. S14F). Furthermore, tofacitinib treatment decreased circulating ABCs in RA patients (Fig. 5J and fig. S14G) and ameliorated systemic inflammation (Fig. 5K). Thus, targeting the JAK–STAT pathway can block ABC differentiation in both mice and humans, making it a promising strategy for the treatment of ABC-mediated autoimmunity.

## Discussion

We have identified ZEB2 as an essential TF that drives human and mouse ABC differentiation, antinuclear antibody formation, proinflammatory cytokine and chemokine production, and ABC migration to inflamed tissues. ZEB2 drives the ABC gene signature including *Itgax,* and suppresses *Mef2b*, which causes activated B cells to deviate from GCs and differentiate extrafollicularly. Differentiation of ABCs in a GC-independent manner has raised questions about the role of GCs in autoimmunity (21, 22). GC reactions comprise several tolerance checkpoints that are lacking in ABC development. Although ZEB2 appears to be essential for ABC formation, the upstream physiological signals and cells that turn on *Zeb2* expression in vivo remain unclear. Zeb2 is likely to act in concert with other transcription or epigenetic factors including IRF5, T-bet, and metabolic regulators, to shape a regulatory complex in ABCs, mirroring ZEB2’s regulatory programs in NK cells (32) and CD8 T cells (33).

Our study highlights the innate ability of ABCs to phagocytose apoptotic cells, a function that may underpin TLR7 activation and self-antigen presentation to T cells, as well as explaining their hyperactivated status. The requirement of the JAK-STAT pathway to exert Zeb2-mediated ABC development and pathogenicity offers promising therapeutic prospects through Jak inhibitors. These insights extend to conditions where ABCs are expanded and may exert pathogenic roles such as aging.

## Supplementary Material

Supplementary material

## Data Availability

All data are available in the main text or the supplementary materials. The scRNA-seq, RNA-seq, ATAC-seq, CUT&Tag, and CUT&RUN sequencing data are deposited in GEO under accession numbers GSE242615, GSE242607, and GSE242611. Plasmids are available upon establishment of an MTA with Shanghai Jiaotong University. All data are available in the main text or the supplementary materials.

## One-Sentence Summary

ZEB2 is essential for age-associated B cell differentiation and function.
