# Supplementary material for: The transcription factor ZEB2 drives formation of age-associated B cells
Source: Science. Author manuscript; Available in PMC 2024 May 29. (PMC7616037; doi:10.1126/science.adf8531)
Supplement: Supplementary material [file EMS196087-supplement-Supplementary_material.pdf]

Science
AAAS

# Supplementary Materials for

## The transcription factor ZEB2 drives the formation of age-associated B cells

Dai Dai *et al*.

Corresponding authors: Carola G. Vinuesa, carola.vinuesa@cric.ac.uk; Nan Shen, nanshensibs@gmail.com

*Science* **383**, 413 (2024)
DOI: 10.1126/science.adf8531

**The PDF file includes:**

Materials and Methods
Figs. S1 to S14
Tables S1 to S9
References

**Other Supplementary Material for this manuscript includes the following:**

MDAR Reproducibility Checklist

## Materials and Methods

### Mice

C57BL/6J (JAX000664), bm12 (JAX001162), *Cd19*-Cre (JAX006785), B6 CD45.1 (JAX002014) and Cas9-EGFP (JAX026175) mice were obtained from Jackson Laboratory. Additionally, *Zeb2*$^{flox/flox}$ mice (T015122) and *Tbx21*$^{tdTomato}$ (T036727) (*26*) mice were obtained from Gempharmatech (Nanjing). All mouse strains were on a C57BL/6J background and maintained under specific pathogen–free (SPF) conditions in Renji Hospital animal facilities. All procedures involving animals were approved by Institutional Animal Care and Use Committee at Renji Hospital of Shanghai Jiaotong University School of Medicine (SJTUSM). All experiments were performed with 8-to-12-week-old male and female mice and using age- and sex-matched groups. Mice were randomly assigned to treatment with JAK–STAT inhibitors. Blinding was used for the evaluation of kidney histopathology and Hep-2 cell autoantibody test.

### Animal models

#### *The bm12 cGVHD lupus model and JAK–STAT-inhibitor treatment*

Female mice were subjected to the bm12 cGVHD lupus model following a previously published protocol (*34*). Briefly, mice were intraperitoneally injected with $1\times10^7$ $CD4^+$ T cells isolated from bm12 mice via magnetic cell sorting (Miltenyi Biotec, Cat#130-104-454). After 2 weeks, mice were sacrificed for analysis. For the JAK–STAT-inhibitor treatment experiments, mice were orally administered with 30 mg of tofacitinib per kilogram of body weight (Selleckchem, Cat#S2789) upon bm12 cGVHD induction for a period of 2 weeks.

#### *Imiquimod (IMQ)-induced lupus model*

Female mice were subjected to the IMQ-induced lupus model following a previously published protocol (*35*). Briefly, mice were treated topically with 1.25 mg of 5% imiquimod cream (3M Health Care Limited) applied on appropriate ear skin areas three times weekly. After 6 weeks, mice were sacrificed for analysis.

#### *4-hydroxy-3-nitrophenylacetyl (NP) hapten immunization model*

Female mice were intraperitoneally administered with 200 µl of a NP-CGG–alum–LPS solution, prepared by adding alum in a dropwise fashion (Imject Alum, Thermo Scientific, Cat#77161) to a solution of 1 mg/ml of NP-conjugated chicken gamma globulin (NP-CGG) (Biosearch Technologies, Cat#N-5055C) and 10 µg/ml of LPS from E. coli 055:B5 (InvivoGen, Cat#tlrl-b5lps) at a 1:1 volumetric ratio. After 14 days, mice were sacrificed for analysis.

#### *LCMV infection*

Female mice were intraperitoneally administered with $1.5\times10^5$ PFU of LCMV-Armstrong, which was kindly provided by J. Li (Fudan University). After 10 days, mice were sacrificed for analysis.

### Study subjects

#### *SLE and RA Patients*

SLE and RA patients were recruited from Renji Hospital. SLE patients met the 1997 American College of Rheumatology criteria for SLE. Demographic and clinical information is listed in table S1 (for scRNAseq) and table S4 (for biopsy samples collected from lupus nephritis patients). RA patients met the 2010 American College of Rheumatology criteria for RA. Clinical and laboratory

data of RA patients, which was measured at baseline and at week 4 after tofacitinib treatment, are listed in table S6.

***MWS patients***

Patients were recruited from Children's Hospital, Capital Institute of Pediatrics. Patients were diagnosed based on clinical evaluation and physical examination findings, and confirmed by genetic testing for mutations in the *ZEB2* gene. Demographic and clinical information are listed in tables S2 and S3.

Written informed consent was obtained from all subjects or their legal guardians. All procedures were conducted with full consent under ethical and safe protocols approved by the Review Board at Renji Hospital of SJTUSM and Children's Hospital, Capital Institute of Pediatrics.

**Cells isolation, culture, and flow cytometry**

***Cell isolation***

Human PBMCs were isolated by density gradient centrifugation using Ficoll-Paque (GE Healthcare, Cat#17-5442-03). Human B cells were isolated by negative selection (Miltenyi Biotec, Cat#130-091-151) from PBMCs. Mouse splenic B cells were isolated by negative selection (Miltenyi Biotec, Cat#130-090-862) according to the manufacturer's instructions.

Mouse kidney tissues were minced and digested with 250 ng/ml of type IV collagenase (Sigma, Cat #C5138) and 10 U/ml of DNase I (Sigma, Cat#D5025) for 30 min at 37°C in a shaking incubator. The digested cells were filtered through a 70-µm nylon mesh and resuspended in 40% Percoll underlaid with 80% Percoll (GE Healthcare Life Sciences, Cat#17-0891-01). Lymphocytes were enriched in the middle layer after density-gradient centrifugation at 2100 rpm for 20 min. The harvested cells were washed two times with wash buffer containing 2% fetal bovine serum (FBS, Gibco, Cat#12483020) and 2 mM EDTA (Invitrogen, Cat#AM9261) in PBS and centrifuged at 400*g* for 5 min.

***Cell culture***

Plat-E cells were cultured in Dulbecco's Modified Eagle Medium (DMEM) with 10% FBS, 1 µg/ml of puromycin (Invivogen, Cat#ant-pr-1), 10 µg/ml of blasticidin (Invivogen, Cat#ant-bl-1), and 100 U/ml of penicillin–streptomycin (Gibco, Cat#15140-122). bEnd.3 cells were cultured in DMEM with 20% FBS. Primary mouse and human B cells were cultured in RPMI-1640 medium with 10% FBS, 2 mM HEPES (Gibco, Cat#15630-080), 1X non-essential amino acids (Gibco, Cat#11140-050), 2 mM glutamine (Gibco, Cat#25030-081), 2 mM sodium pyruvate (Gibco, Cat#11360-070), 55 µM β-mercaptoethanol (Gibco, Cat#21985-023), and 100 U/ml of penicillin–streptomycin. Mouse B cells were induced for in vitro ABC differentiation in the presence of mouse ABC-skewing cocktail containing 500 ng/ml of R848 (Invivogen, Cat#tlrl-r848-5), 1 µg/ml of anti-CD40 (Biolegend, Cat#102812), 1 µg/ml of anti-IgM $F(ab')_2$ (Jackson Immunoresearch, Cat#115-006-020), 50 ng/ml of IL-21 (Peprotech, Cat#210-21) , and 1 ng/ml of IFN-γ (Biotech, Cat#485-MI-100) or at the indicated final concentrations for 3 days. Human B cells were stimulated with human ABC-skewing cocktail containing 1 µg/ml of R848, 10 µg/ml of CD40L (Biolegend, Cat#591708), 20 ng/ml of BAFF (Peprotech, Cat#310-13-20), 10 ng/ml of IL-2 (Peprotech, Cat#200-02), 10 µg/ml of goat anti-human IgA + IgG + IgM (H+L) (Jackson Immunoresearch, Cat#109-006-064), 10 ng/ml of IL-21 (Biolegend, Cat#571204), and 20 ng/ml

of IFN-γ (Biolegend, Cat#570206) for 3 days. The JAK–STAT inhibitors tofacitinib or baricitinib (Selleckchem, Cat#S2851) were added in the B cell culture at indicated working concentrations and incubated for 3 days. All cells were cultured in a humidified 37°C incubator set at 5% $CO_2$.

***Flow cytometry***
Cells were stained for surface markers or intracellular proteins according to published guidelines (*36*). To screen the functional transcriptional factor for mouse ABC formation, B cells were stained with surface markers and then fixed by 2% fresh prepared formaldehyde in PBS for 5 min to allow efficient staining of transcription factors (TF) and retaining of fluorescent proteins which were optimized from the previous protocol (*37*). For intracellular T-bet staining, cells were fixed and permeabilized after surface staining using Foxp3 Transcription Factor Staining Buffer Set (ThermoFisher, Cat#00-5523-00). Data were acquired using LSRFortessa (BD Biosciences) and analyzed using FlowJo software (Tree Star). Cell sorting were conducted by FACSAria II (BD Biosciences). Gating strategies for flow cytometry plots are shown in figs. S2, A and B, S3, B and C, S5, A and C, S6E, S8, D and G, and S14G. Detailed information for antibodies used is listed in table S7.

***t-SNE visualization of flow cytometric data***
All samples were pregated on live single lymphocytes or B cells. Each sample was randomly down sampled with the same cell number and merged into a single expression matrix. The t-SNE (t-distributed stochastic neighbor embedding) was applied to reduce the dimensionality of the data using the t-SNE plugin available on the FlowJo Exchange. The composite sample then was hand-gated as indicated for all populations to aid in visual overlays with t-SNE maps. Intensities for markers of interest were overlaid on the t-SNE maps to show the expression of those markers on different cell clusters.

**Immunohistochemistry**
The kidneys from IMQ-induced lupus mice were fixed in 4% neutral buffered formalin and embedded in paraffin. Tissue sections with a thickness of 5 μm were stained with H&E and were scanned using Pannoramic MIDI Digital Section Scanner (3DHistech). The kidney pathologic score was accounted for the morphological pattern (glomerular mesangial cells and inflammatory cellular infiltration) using Case Viewer software (3DHistech).

**In vitro apoptotic cell phagocytosis assay**
Thymocytes were isolated from C57BL/6 mice and cultured in RPMI-1640 medium supplemented with 10% FBS, 100 U/ml of penicillin–streptomycin, and 500 nM camptothecin (Selleck, Cat#S1288) for 24 hours to induce apoptosis. The frequency of apoptotic cells was confirmed above 80% via apoptosis detection kit (Biolegend, Cat#640932).

For detection by flow cytometry, apoptotic thymocytes were stained with 60 ng/ml of pHrodo (Invitrogen, Cat#P35357) for 2 hours at 37°C. The labeled apoptotic thymocytes were then coincubated with target cells mixed at a 10:1 apoptotic-thymocyte-to-target-cell ratio in the presence of R848 (1 μg/ml). After incubation for 2 hours, phagocytosis was assessed.

For real-time detection, apoptotic thymocytes were stained using the IncuCyte pHrodo Red Cell Labeling Kit (Sartorius, Cat#4649). Briefly, apoptotic thymocytes were washed with provided 1X wash buffer and resuspended in cell labeling buffer at a concentration of $1\times10^6$ cells/ml.

pHrodo dye was then added to a final concentration of 100 ng/ml and cells were stained for 60 min at 37°C. The labeled apoptotic thymocytes were coincubated with target cells in 24-well-plate at a 10:1 apoptotic-thymocyte-to-target-cell ratio in the presence of R848 (1 µg/ml). The plate was then transferred to the Incucyte Live-Cell Analysis System for images capture for 4 hours and each well was imaged every 30 min.

**In vitro transwell migration assay and transendothelial migration assay**
B cells were resuspended in migration medium (RPMI 1640 supplemented with 0.5% fatty acid–free BSA, 100 U/ml of penicillin–streptomycin, and 2 mM HEPES). Transwell inserts (Corning; Cat#CLS3421) were placed over each well. Cell suspensions were added into the upper chamber and allowed to migrate for 6 hours. Migrated cells in the lower chamber were collected and stained for detection by flow cytometry. B cells were pretreated with 25 µg/ml of anti-mouse CD11c Antibody (Biolegend, Cat#117302) for 1 hour before the effects of CD11c blockade were measured.

The Transwell inserts were coated with Matrigel (Corning, Cat#356324) for 30 min at 37°C. Murine endothelial cells (bEnd.3) were then seeded onto the Matrigel-coated Transwell inserts for 24 hours to form a compact monolayer. B cells were subsequently added to the upper chamber and 100 ng/ml of CXCL12 (Peprotech; Cat#25020B) was added to the lower chamber. After incubation for 6 hours at 37°C, the migrated cells were collected from the lower chamber and washed two times with PBS supplemented with 2% FBS and 2 mM EDTA with centrifugation at 400*g* and stained for detection by flow cytometry.

**Vector construction, retrovirus packaging, and infection**
***Vector construction***
The sgRNA expressing LMP-U6-sgRNA(BbsI)-PGK-Puro-T2A-BFP vector was generated by cloning the PCR-amplified U6-sgRNA(BbsI)-PGK-Puro-T2A-BFP fragment from pKLV-U6-gRNA(BbsI)-PGK-puro-2A-BFP plasmid (Addgene, Cat#50946) into SalI and XhoI sites of the LMP retroviral vector (Open BioSystems, Cat#EAV4678). sgRNA sequences were designed by online design tool (*38*). The paired forward and reverse oligos were phosphorylated and annealed by T4 Polynucleotide Kinase (NEB, Cat#M0201). Then annealed oligo duplexes were cloned into the BbsI sites of the LMP-U6-sgRNA(BbsI)-PGK-Puro-T2A-BFP vector by T4 DNA ligation (NEB, Cat#M0202). To overexpress ZEB2 and T-bet, the cDNA sequence of *Zeb2* and *Tbx21* were synthesized and inserted into the PacI and Not1 site of pMYs-IRES-GFP retroviral vector (Cell Biolabs, Cat#RTV-021). The inserted sgRNA and cDNA sequences were validated by Sanger sequencing.

***Retrovirus packaging***
Five million Plat-E cells were plated in a 10-cm dish in DMEM with 10% FBS. Cells were transfected 1 day later with 15 µg of sgRNA plasmid using 30 µl of Lipofectamine 2000 (Invitrogen, Cat#11668019). Plat-E cells were next cultured for another 48 hours. The retrovirus supernatants were then harvested and then either filtered using a 45-µm filter or centrifuged at 500*g* for 5 min to remove cells and debris.

***Retrovirus infection***

Splenic B cells from C57BL/6J or Cas9-EGFP mice were activated in the presence of 500 ng/ml of R848, 1 µg/ml of anti-CD40, and 1 µg/ml of anti-IgM $F(ab')_2$ for 1 day. Then retroviruses were spin-transduced at 1260*g* for 90 min at 32℃ in the presence of 10 µg/ml of polybrene (Sigma, Cat#H9268) to infect B cells. The medium was then removed 6 hours later and replaced with fresh medium. The infected cells were further cultured in the presence of mouse ABC-skewing cocktail as described above for 3 days. At least four sgRNA plasmids were constructed per target gene and editing efficiency was validated in mouse primary B cells using the online inference of CRISPR edits (ICE) tool (*39*). The most efficient sgRNA for each target gene with an editing rate over 50% (except for sg-*Plek* 46%) was used for screening. The selected sgRNA sequences and editing efficiency in primary mouse B cells are listed in table S8.

**Human B cell editing**

Purified B cells from human PBMC were stimulated with 1 µg/ml of R848, 10 ng/ml of IL-2, 10 ng/ml of BAFF, and 10 µg/ml of goat anti-human IgA + IgG + IgM (H+L) for 1 day. Synthetic sgRNA oligos (GenScripts) were incubated 1 day later with Cas9 protein (GenScripts, Cat#Z03389) at room temperature for 20 min. One hundred thousand cells were washed and electroporated using the Neon transfection system (Thermofisher, Cat#MPK5000S) following the manufacturer's instructions. For each target gene, we validated the efficiency of sgRNA with an editing rate over 50% (except for sg-*ETS1* 46% and sg-*JUN* 33%). The sgRNA sequences and editing efficiency were listed in table S8. After electroporation, human B cells were further cultured for 3 days in the presence of human ABC-skewing cocktail as described above.

**Antibody titers, autoantigen array and multiplex immunoassay**

Blood sera were collected at indicated time points from IMQ-induced and bm12-induced lupus models. The supernatants were collected from the ex vivo culture of sorted cells from the IMQ-induced mice. Two hundred thousand sorted cells were seeded per well in a 96-well plate and stimulated in the presence of 500 ng/ml of R848, 1 μg/ml of anti-CD40, and 50 ng/ml of IL-21 with or without 10 ng/ml of IFN-γ for 24 hours.

Anti-dsDNA antibody titers in the serum were detected by ELISA (Alpha Diagnostic International, Cat#5110). Anti-nuclear antibodies (ANA) titers in the serum were captured in Hep-2 cells slides (Inova diagnostics, Cat#708750) and used Alexa Fluor 488–conjugated anti-mouse IgG (Biolegend, Cat#405310) for immunofluorescence. Immunofluorescence images were captured using Olympus IX73 or Leica DMI 3000B fluorescence microscope with a 10X objective lens. Antibodies in the culture supernatants were captured by goat anti-mouse Ig (Southern Biotechnology, Cat#5300-05B) and detected by HRP-labeled goat anti-mouse IgG1-, IgG2c-, IgG3-, or IgM-specific antibodies and TMB substrate (Biotech, Cat#DY999) followed by sulfuric acid stop. Absorbance values were read at 370/450 nm (OD). Where appropriate, the absolute titers were calculated from standard curves, which were generated from purified mouse Ig isotype (Southern Biotechnology, Cat#5300-01B). The autoantibody profiling of the serum samples was measured using an Autoantigen Microarray platform developed by the Genomics and Microarray Core Facility, UT Southwestern Medical Center. The concentrations of cytokine and chemokine in the culture supernatants were detected using the Bio-Plex Pro Mouse Chemokine Panel 31-Plex (Bio-Rad, cat#12009159) according to the manufacturer's instructions.

**RNA extraction and quantitative RT-PCR analysis**

Total RNA was extracted using TRIzol reagent (ThermoFisher, Cat#15596026). RNA was assessed using the Nanodrop 2000. cDNA was synthesized using the PrimeScript RT Reagent Kit (TaKaRa, Cat#RR037A）. Real-time PCR were conducted using TB Green Premix Ex Taq reagent (TaKaRa, Cat#RR420A) on QuantStudio 7 Flex (Applied Biosystems). For quantification of gene expression, each sample was normalized to expression of an endogenous control gene. All primers used are listed in table S9.

**Single-cell RNA library preparation and sequencing**

$CD19^+$ B cells isolated by flow cytometry from PBMCs of a patient with new-onset SLE were resuspended in PBS. The viability was confirmed to be >90%. Single-cell RNA libraries were prepared and sequenced on an Illumina NovaSeq 6000 platform as previously described (*40*). Briefly, single-cell suspensions were loaded on a 10x Genomics Chromium single-cell 5′ v2 chip. After capture and lysis, cDNA was synthesized and amplified according to the manufacturer's protocol. The library quality was assessed with an Agilent 2100 Bioanalyzer and then sequenced with a depth of >50,000 reads per cell.

**Processing of single-cell RNA-seq data**

The raw FASTQ data were processed with Cell Ranger (v3.1.1) (*41*) by using GRCh37 annotation. Output from CellRanger was loaded into R and analysed by Seurat package (v3.1.1) (*42*). A total of 5721 cells with <500 or >5000 expressed genes or a high percentage of mitochondrial genes (>10%) were removed. The UMI counts in each cell were then normalized and scaled. The top 1500 highly variable genes were used for dimensionality reduction using principal components analysis. We selected the first 15 principal components for clustering according to the result from the elbow method and used UMAP visualization with default parameters. For clustering, we first selected resolution parameter 0.8 which produced nine clusters. To obtain cluster-specific gene signatures, we identified differential expression analysis of each cluster against the others using wilcox with parameter (logfc. threshold 0.25, min.pct 0.1). Clusters were manually merged with the nearest cluster based on the phylogenetic tree from Seurat's Build Cluster Tree and similar marker genes. We ultimately obtained seven clusters for downstream analysis.

**Bulk RNAseq**

***RNA preparation***

Splenic B cells from C57BL/6J mice were retrovirally transduced with pMYs-IRES-GFP or pMYs-*Zeb2*-IRES-GFP plasmids. Splenic B cells from Cas9-EGFP mice were retrovirally transduced with sg-NC, sg-*Zeb2*, or sg-*Tbx21* plasmids. The infected cells were further cultured in the presence of mouse ABC-skewing cocktail as described above for 3 days. Cells were stained with anti-mouse CD19 and $CD19^{+}EGFP^{+}$ (for overexpression) or $CD19^{+}EGFP^{+}BV421^{+}$ cells were isolated by FACS. The harvested cells were washed twice in PBS by centrifugation at 400*g* for 5 min. RNA was extracted with RNeasy Micro kit (Qiagen, Cat#74004) for *Zeb2* overexpressed B cells or TRIzol reagent.

***cDNA Library construction and sequencing***

RNA was assessed with an Agilent 2100 Bioanalyzer (RNA integrity number >7 for all samples) and used as input for TruSeq RNAseq library kit (Illumina, Cat#RS-122-2001) or SMART-Seq v4 Ultra Low Input RNA kit for Sequencing (Takara #634888). After quality assessment, cDNA libraries were pooled and processed by an Illumina NovaSeq 6000 sequencer for 150-bp paired-end sequencing.

***Data analysis***

Clean reads were aligned to GRCm38 reference genome with Hisat2 (v2.1.0) (*43*) using default parameters. Gene expression levels were counted with HT-seq (v0.11.2) (*44*) using default "union" mode. Differential expressions of genes were conducted by DESeq2 (v1.24.0) package (*45*) and simple plots were produced in R (v3.3.3). GSEA was performed using the online software (Broad Institute).

**ATAC-seq and data analysis**

***Sample preparation***

$CD11c^{+}$T-$bet^{+}$ and $CD11c^{-}$T-$bet^{-}$ B cells were sorted by flow cytometry from bm12-induced T-bet reporter mice. Splenic B cells from B-*Zeb2*$^{KO}$ and *Cd19*-cre mice were cultured in the presence of mouse ABC-skewing cocktail as described above for 3 days and were purified using Dead Cell Removal Microbeads (Miltenyi Biotec, Cat#130-090-101). The harvested cells were resuspended in lysis buffer (10 mM Tris-Cl, 10 mM NaCl, 3 mM $MgCl_2$, and 0.1% Igepal CA-630) and incubated on ice for 10 min following centrifugation at 500*g* for 5 min at 4°C.

***Library construction***

DNA library for ATAC-seq was performed using TruePrep DNA Library Prep Kit V2 for Illumina (Vazyme, Cat#TD501). Briefly, The cell pellet was resuspended in transposase reaction mix and incubated at 37℃ for 30 min. Fragmented DNA was purified using VAHTS DNA Clean Beads (Vazyme, Cat#N411) and library was generated by PCR using TruePrep Index Kit v2 for Illumina (Vazyme, Cat#TD202) for 11 cycles. PCR cleanup of libraries was performed using VAHTS DNA Clean Beads at a 1:1.2 ratio. Libraries were then sequenced on Illumina NovaSeq 6000 with paired-end reads.

***Data analysis***

Clean reads were aligned to GRCm38 reference genome using Bowtie2 (v2.3.5) (*46*) with the default setting followed by removing PCR duplicates using Sambamba (*47*). Peaks were called using Genrich with parameter -FDR 0.01. For track display, alignments were converted to bigwig file using bedtools (*48*).

**CUT&RUN**

CUT&RUN libraries were generated following manufacturer's instructions (CST, Cat#86652). Briefly, $CD19^{hi}CD11c^{+}CD21^{-}$ cells were sorted from IMQ-induced lupus mice and incubated with Concanavalin A Magnetic Beads. Cells were permeabilized and then incubated with primary antibodies against ZEB2 (Novus Biologicals, Cat#NBP1-82991) for 2 hours followed with Goat Anti-Rabbit IgG secondary antibody (Abcam, Cat#ab6702) for 1 hour. This mixture of beads and cells was resuspended in pAG-MNase Enzyme and incubated for 1 hour followed with activation of enzyme by adding calcium chloride at 4°C for 30 min. DNA was then extracted using phenol–chloroform–isoamyl alcohol as the input. The libraries were prepared with the NEBNext Ultra II DNA Library Prep Kit for Illumina (NEB, Cat#E7645) according to the manufacturer's instruction. Libraries with different indexes from NEBNext Multiplex Oligos for Illumina (NEB, #E7335) were pooled and sequenced with an Illumina Hiseq PE150.

Sequencing data were analyzed as follows. The adaptor sequences were discarded by Trimmomatic before alignment. To visualize CUT&RUN datasets with UCSC genome browser, the CUT&RUN reads were aligned and mapped to the mouse reference genome (GRCm38/mm10) by Bowtie2 (v2.3.5) software (*46*). Duplicated reads were removed using 'make tag directory' in HOMER (v4.11) with the parameter -tbp 1. The makeUCSCfile in Homer was used for visualization in UCSC genome browser.

**CUT&Tag**

Splenic B cells from C57BL/6J mice were cultured in the presence of mouse ABC-skewing cocktail for 3 days and were purified using Dead Cell Removal Microbeads. CUT&Tag libraries were generated following instructions of manufacturer's protocol (Vazyme, Cat#TD901). Briefly, the harvested cells were incubated with Concanavalin A Magnetic Beads. Cells were permeabilized and then incubated with primary antibodies against ZEB2 (Novus Biologicals, Cat#NBP1-82991) for 2 hours followed with goat anti-rabbit IgG secondary antibody (Abcam, Cat#ab6702) for 1 hour in Dig-Wash Buffer at room temperature. The 0.04 μM pG-Tn5 transposase were added for tagmentation at room temperature for 1 hour. DNA was then extracted using phenol–chloroform–isoamyl alcohol. Libraries were prepared and cleaned up using Ampure XP beads (Beckman Coulter, Cat#A63880) and pooled together for paired-end sequencing.

Sequencing data were analyzed as follows. After trimming the adapters of reads and removing low-quality bases by TrimGalore software in paired-end mode, clean reads were mapped to mm10 reference genome with Bowtie2 (v2.3.5) (*46*) followed by removing PCR duplicates using Picard (v2.19.0). The genome browser tracks in bigwig format were produced from merged replicates using deepTools (v2.0) (*49*) and peaks were called using MACS2 (v2.1.2) (*50*) using parameters -f BAMPE –SPMR -nomodel. Enriched transcription factor binding motifs were searched by HOMER (v4.11). A set of sequences were searched for individual matches to each of the motif using FIMO (*51*). All analysis data were visualized using the UCSC genome browser.

Intersect in bedtools was used to screen for overlaps among peaks from ATAC-seq of CD11c$^+$T-bet$^+$ B cells, ZEB2 CUT&RUN, and ZEB2 CUT&Tag as described above. Annotation of the overlapping peaks were performed with ChIPseeker.

**IPA analysis**

To define the distinct biological function and upstream regulators of ABCs, we integrated the public datasets and our own datasets to perform core analysis using ingenuity pathway analysis (IPA) software. The cut-off for ABC specific genes from different settings was set as expression $\log_2$-fold change $\geq 1$ or $\leq -1$ and statistically significant FDR value <0.05. The cut-off for sg-*Zeb2* versus sg-NC was a statistically significant FDR value of <0.05. The significance and regulatory effects of enriched pathways, upstream regulators and biological functions was qualified using overlap *P*-value and activation Z-score based on the observed pattern of up- or downregulation of the target molecules compared with expected directions of changes documented in Ingenuity's curated database.

**Statistical analysis**

Prism software (GraphPad) was used for statistical analyses. Data are displayed as means ± SEM. All data points represented the measurement of distinct samples. Statistical tests were selected based on the distribution and the variance characteristics of the data and indicated in the figure legends. *P*-values were calculated using unpaired two-tailed Student's *t* test and with Welch's correction when necessary, Mann–Whitney *U* test, or one-way ANOVA followed by Dunnet's test. A paired Student's *t* test was used to compare matched samples in Fig. 5, J and K. *P*-values of less than 0.05 were considered significant.

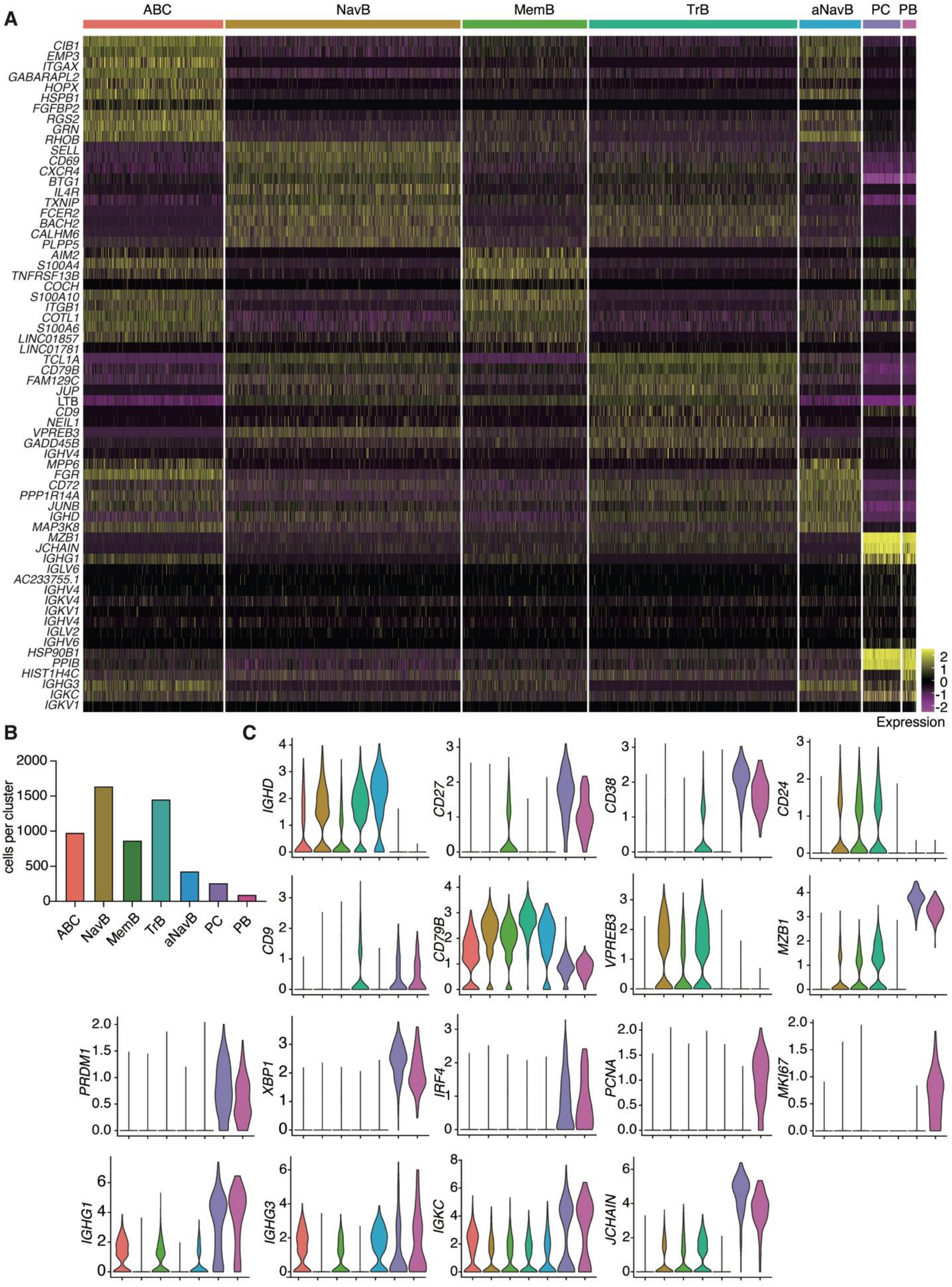
A
ABC
NavB
MemB
TrB
aNavB
PC
PB
CIB1
EMP3
ITGAX
GABARAPL2
HOPX
HSPB1
FGFBP2
RGS2
GRN
RHOB
SELL
CD69
CXCR4
BTG1
IL4R
TXNIP
FCER2
BACH2
CALHM6
PLPP5
AIM2
S100A4
TNFRSF13B
COCH
S100A10
ITGB1
COTL1
S100A6
LINC01857
LINC01781
TCL1A
CD79B
FAM129C
JUP
LTB
CD9
NEIL1
VPREB3
GADD45B
IGHV4
MPP6
FGR
CD72
PPP1R14A
JUNB
IGHD
MAP3K8
MZB1
JCHAIN
IGHG1
IGLV6
AC233755.1
IGHV4
IGKV4
IGKV1
IGHV4
IGLV2
IGHV6
HSP90B1
PPIB
HIST1H4C
IGHG3
IGKC
IGKV1
Expression
B
cells per cluster
C
IGHD
CD27
CD38
CD24
CD9
CD79B
VPREB3
MZB1
PRDM1
XBP1
IRF4
PCNA
MKI67
IGHG1
IGHG3
IGKC
JCHAIN

**Fig. S1. B cell landscape from a new-onset SLE patient. (A)** Heat map representing scaled expression values of top 10 genes defining each cluster. **(B)** Number of cells per cluster. **(C)** Violin plots of select gene expression (*IGHD*, CD27, *CD38*, *CD24*, *CD9*, *CD79B*, *VPREB3*, *MZB1*, *PRDM1*, *XBP1*, *IRF4*, *PCNA*, *MKI67*, *IGHG1*, *IGHG3*, *IGKC*, and *JCHAIN*) in different clusters, with log-normalized expression values labeled.

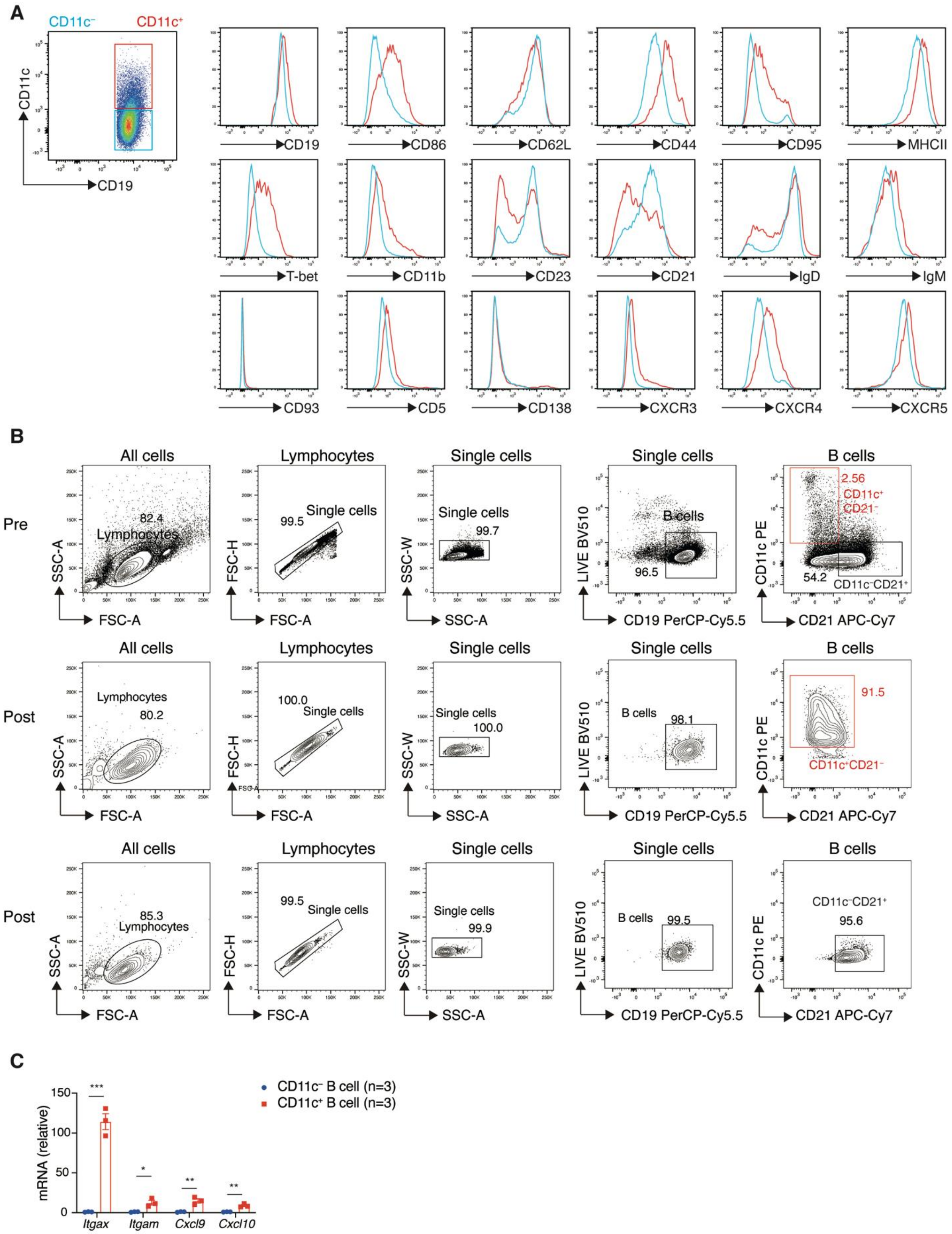


**Fig. S2. Phenotype of ABCs from cGVHD lupus mice. (A)** Flow cytometry detection of characteristic markers of splenic CD11c$^{-}$ (blue) or CD11c$^{+}$ (red) B cells (n=3) from bm12-induced

lupus mice. (**B**) Pre- and post-sort flow cytometry gating of splenic $CD19^{+}CD11c^{+}CD21^{-}$ and $CD19^{+}CD11c^{-}CD21^{+}$ B cells from mice described in (A). (**C**) Real-time PCR analysis of ABC signature genes (*Itgax*, *Itgam*, *Cxcl9*, and *Cxcl10*) in splenic $CD19^{+}CD11c^{+}CD21^{-}$ and $CD19^{+}CD11c^{-}$ $CD21^{+}$ B cells (n=3) sorted from mice as described in (A). n represents distinct samples (biological repeats). Data are representative of two (A) and three (B and C) independent experiments with three mice per group. Bars indicate mean ± SEM values. Statistical significance was analyzed using an unpaired Student's *t* test (C). *$P<0.05$, **$P<0.01$, ***$P<0.001$, ns, not significant.

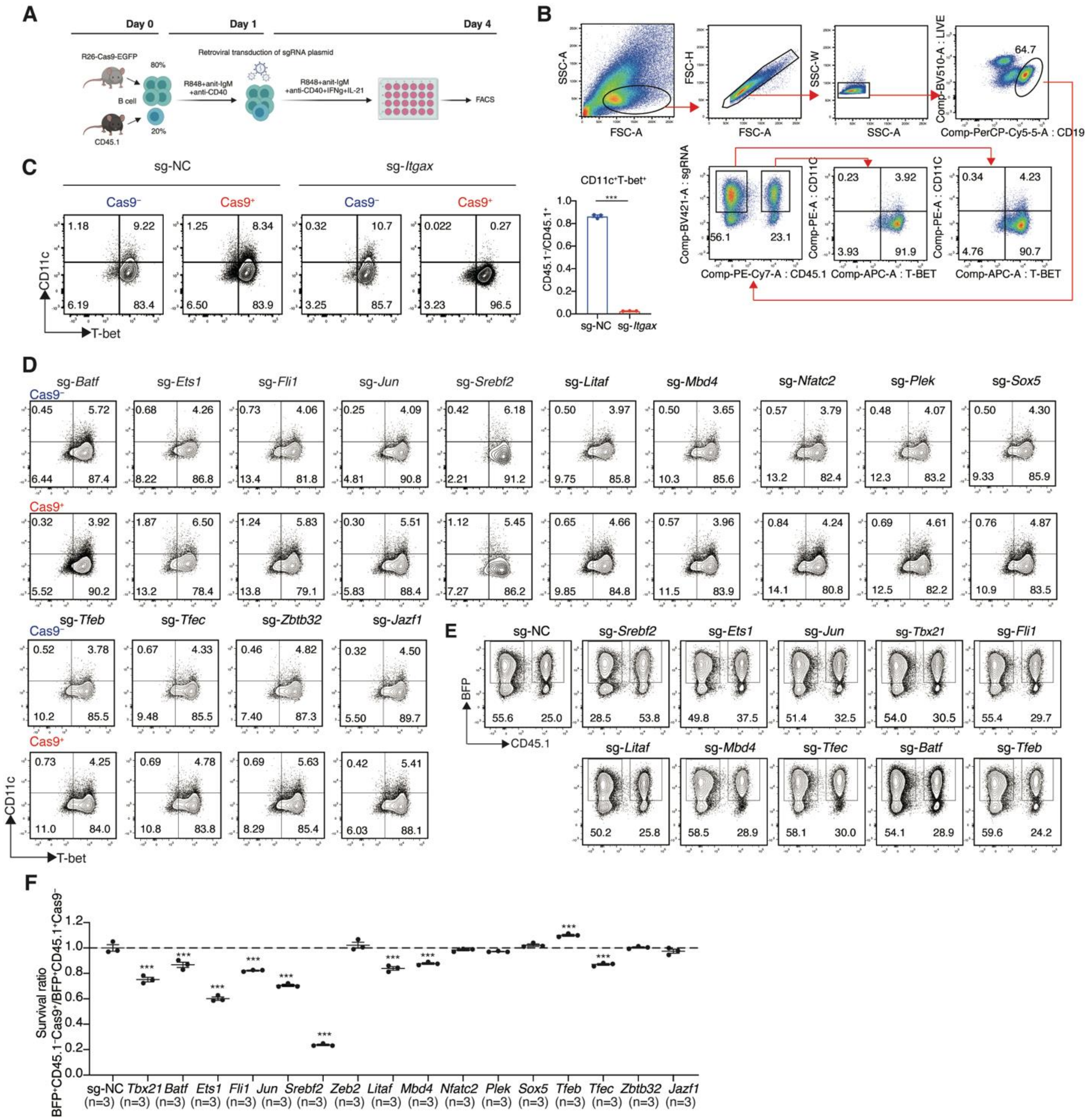

**Fig. S3. Gene editing in mouse primary B cells and screen TFs for ABC differentiation.** (**A**) Flow chart of Cas9 based screen and in vitro mouse ABC differentiation assay. (**B**) Gating strategy for flow cytometry analysis of (A). The ABC percentage in BFP$^{+}$CD45.1$^{-}$ fraction versus percentage in BFP$^{+}$CD45.1$^{+}$ fraction was set and then sg-TF group was normalized with sg-NC group and calculated as ABC ratio. (**C**) Flow cytometry plots and ratio of ABC frequency in CD45.1$^{+}$sg-*Itgax*$^{+}$ (Cas9$^{-}$) and CD45.1$^{-}$sg-*Itgax*$^{+}$ (Cas9$^{+}$) cells. (**D**) Flow cytometry plots of CD11c$^{+}$T-bet$^{+}$ B cells in CD45.1$^{+}$sgRNA$^{+}$ (Cas9$^{-}$sgRNA$^{+}$) and CD45.1$^{-}$sgRNA$^{+}$ (Cas9$^{+}$sgRNA$^{+}$) cells targeting indicated genes. (**E**) Flow cytometry plots of CD45.1$^{-}$ (Cas9$^{+}$) and CD45.1$^{+}$ (Cas9$^{-}$) B cells targeting indicated genes after gating on BFP$^{+}$ cells. (**F**) Statistical analysis of cell survival ratio by comparing CD45.1$^{-}$ (Cas9$^{+}$) with CD45.1$^{+}$ (Cas9$^{-}$) B cells and then normalized with sg-NC. Data are representative of four independent experiments. Bars indicate mean ± SEM values. Statistical significance was analyzed

using unpaired Student's *t* test (C) and ordinary one-way ANOVA with two-sided Dunnett's multiple comparisons testing (F). *$P$<0.05, **$P$<0.01, ***$P$<0.001, ns, not significant.

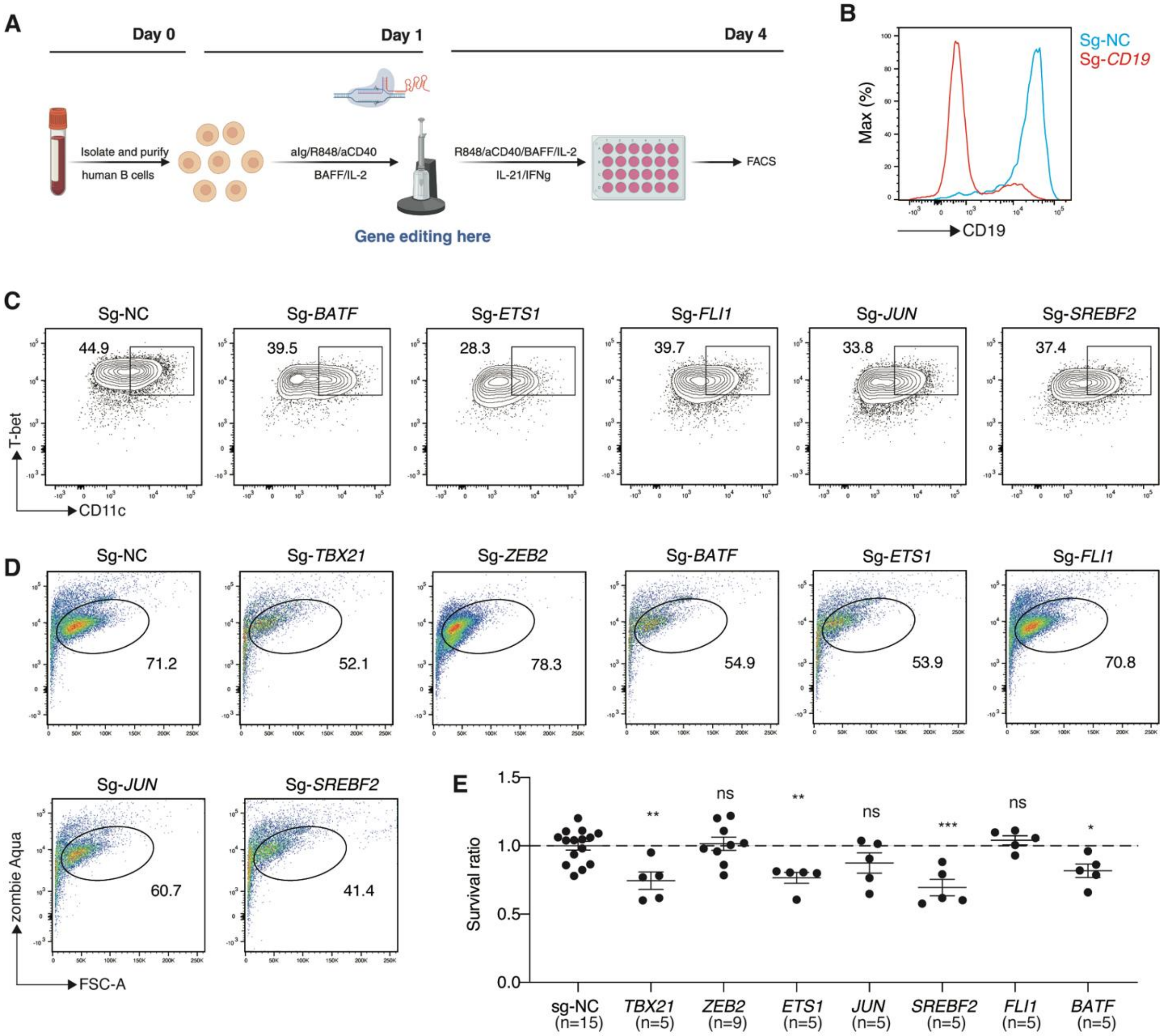


**Fig. S4. Screen TFs for human ABC differentiation.** (**A**) Flow chart of Cas9-gRNA (RNP) complex based screen and in vitro human ABC differentiation assay. (**B**) The decreased CD19 expression by gene editing targeting *CD19* in human primary B cells. (**C**) Flow cytometry plots of human ABCs after gene editing targeting indicated TFs. (**D** and **E**) Flow cytometry plots (D) and ratio (E) of cell viability of B cells electroporated with RNP complex targeting indicated gene. n refers to the measurement of distinct samples (biological repeats). Data are representative of three independent experiments. Bars indicate mean ± SEM values. Statistical significance was analyzed using ordinary one-way ANOVA with two-sided Dunnett's multiple comparisons testing (E). **P*<0.05, ***P*<0.01, ****P*<0.001, ns, not significant.

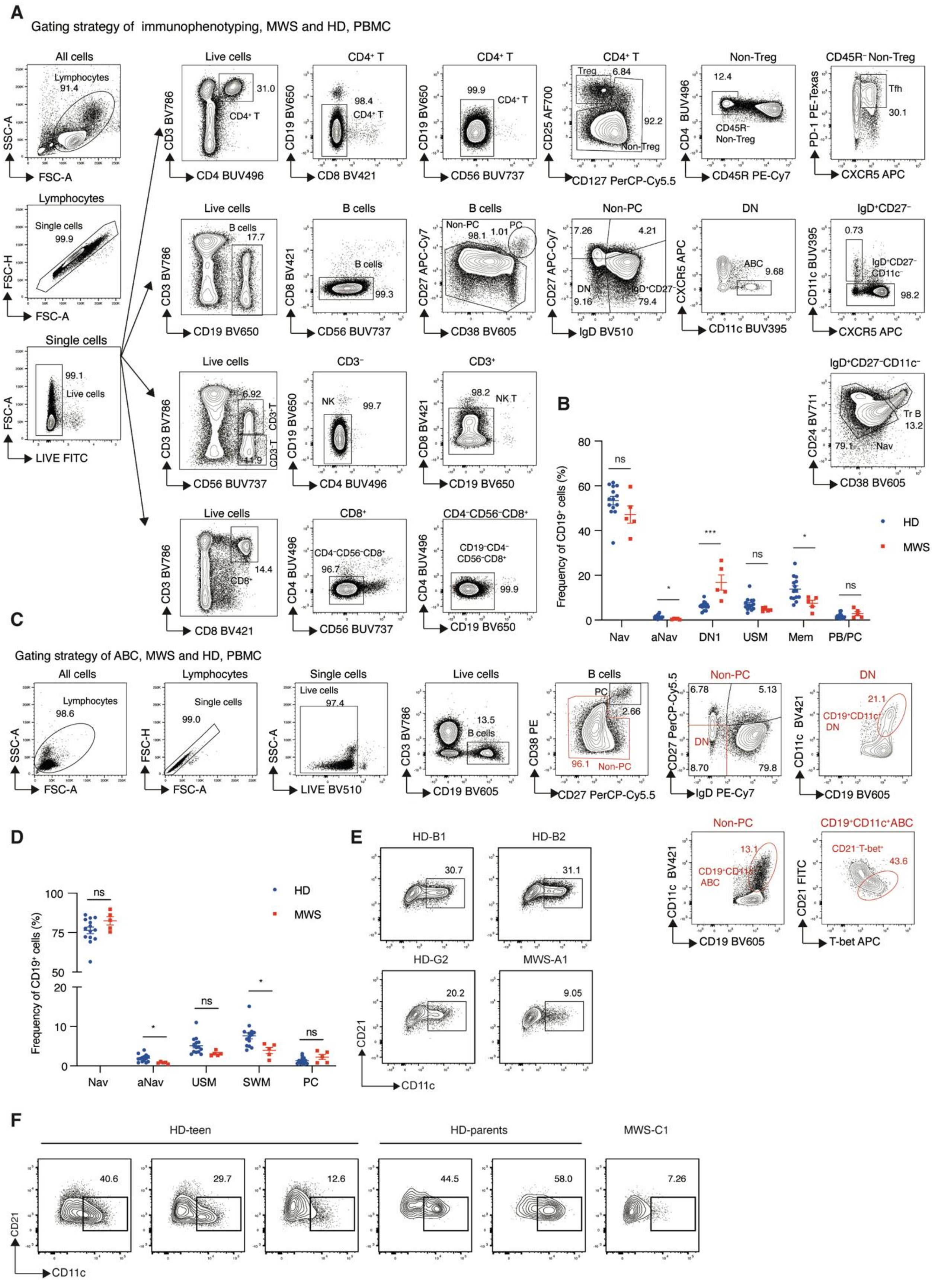
A
Gating strategy of immunophenotyping, MWS and HD, PBMC
B
C
Gating strategy of ABC, MWS and HD, PBMC
D
E
F
HD-teen
HD-parents
MWS-C1
HD
MWS
Frequency of CD19⁺ cells (%)
Nav
aNav
DN1
USM
Mem
PB/PC
SWM
PC
CD21
CD11c

**Fig. S5. Immunological phenotype of MWS patients.** (**A**) Gating strategy for lymphocytes staining in Fig. 2C. **(B)** tSNE analysis of the frequency of Nav, aNav, DN1, USM, Mem, PB/PC in B cells. (**C**) Gating strategy for DN cells ($CD19^{+}CD38^{-}CD27^{-}IgD^{-}$), DN2 cells ($CD19^{+}CD38^{-}CD27^{-}IgD^{-}CD11c^{+}$), $CD11c^{+}$B cells ($CD19^{+}CD38^{lo/mid}CD27^{lo/mid}CD11c^{+}$), and ABCs ($CD19^{+}CD38^{lo/mid}CD27^{lo/mid}IgD^{-}CD11c^{+}CD21^{-}$T-bet$^{+}$) in PBMCs in Fig. 2G. (**D**) Frequency of naive, aNav, USM, SWM, plasma cells in B cells as described in **(**C**).** (**E** and **F**) Representative plots for in vitro–induced ABCs ($CD19^{+}IgD^{-}CD27^{-}CD21^{-}CD11c^{+}$) derived from B cells of MWS patients. n refers to the measurement of distinct samples (biological repeats). Bars indicate mean ± SEM values. Statistical significance was analyzed using unpaired Student's *t* test (B and D). * $P<0.05$, ** $P<0.01$, ***$P<0.001$, ns, not significant.

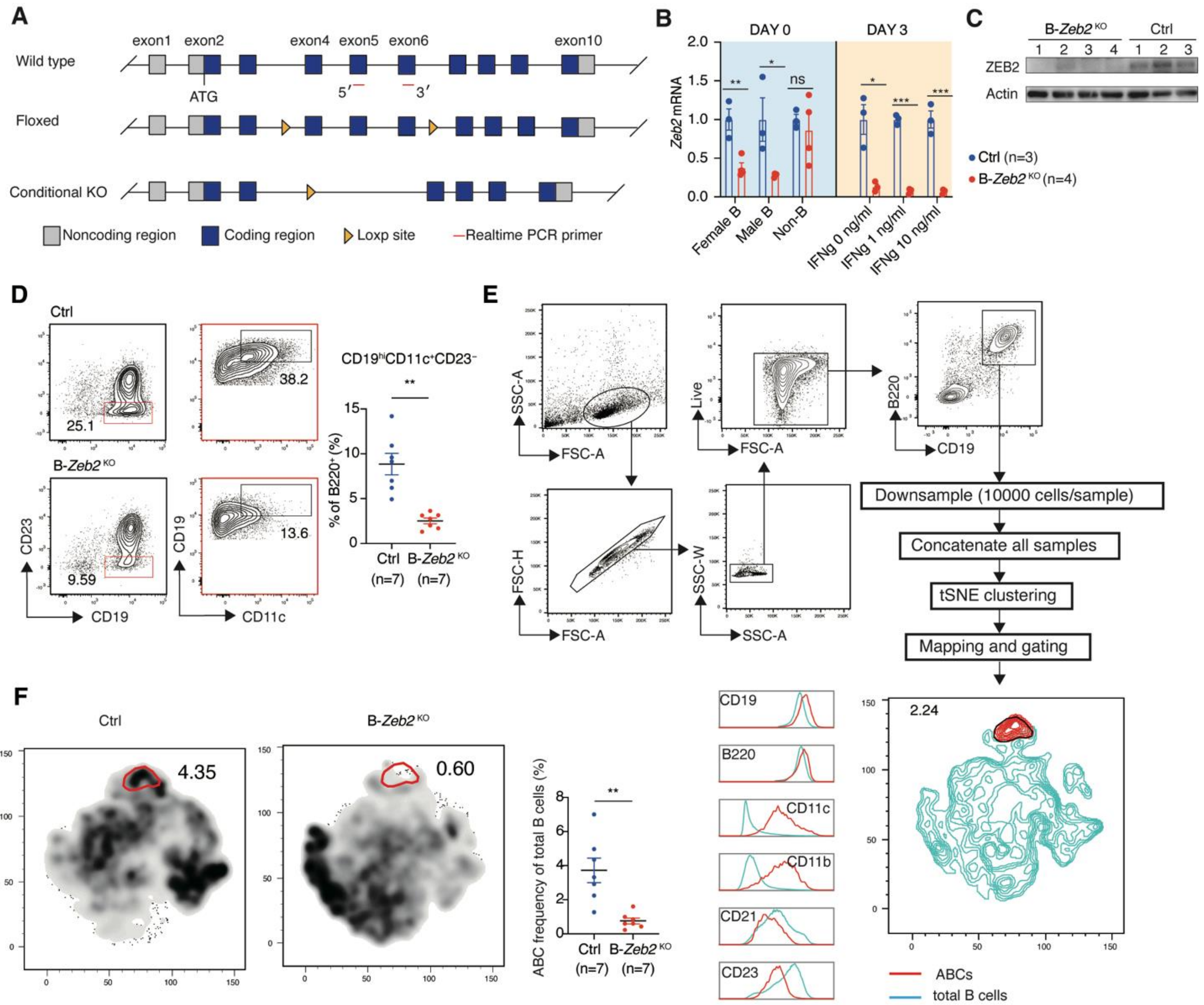


**Fig. S6. Construction of B-*Zeb2*$^{KO}$ mice and IMQ-induced lupus model.** (**A**) Schematic diagram for construction of *Zeb2* conditional knockout allele. Exons 4 to 6 were chosen as a target for inserting Loxp sites by using CRISPR/Cas9 technology. The *Zeb2* gene was ablated after crossing with mice expressing Cre recombinase. Real-time PCR primers to detect *Zeb2* expression are labeled in red. (**B**) Real-time PCR analysis of *Zeb2* expression in splenic B cells and non-B cell fractions (Day 0) or ABC-skewing cocktail for 3 days. (**C**) Immunoblot analysis of ZEB2 protein level in splenic B cells from B-*Zeb2*$^{KO}$ and *Cd19*$^{Cre/+}$ (Ctrl) mice. (**D**) Flow cytometry plots and frequency of splenic ABCs (CD19$^{hi}$CD11c$^{+}$CD23$^{-}$) from B-*Zeb2*$^{KO}$ and *Cd19*$^{Cre/+}$ (Ctrl) mice as described in Fig. 3C. (**E**) Analysis strategy and t-SNE plots of composite splenic B cell samples from IMQ-induced B-*Zeb2*$^{KO}$ and Ctrl mice. (**F**) Separated t-SNE plots for two representative B-*Zeb2*$^{KO}$ (left) and Ctrl (right) samples. Frequency of ABC in IMQ-induced B-*Zeb2*$^{KO}$ versus control mice. n refers to the measurement of distinct samples (biological repeats). Data are representative of two independent experiments. Bars indicate mean ± SEM values. Statistical significance was analyzed using unpaired Student's *t* test (B) with Welch's correction (D and F). **P*<0.05, ***P*<0.01, ****P*<0.001, ns, not significant.

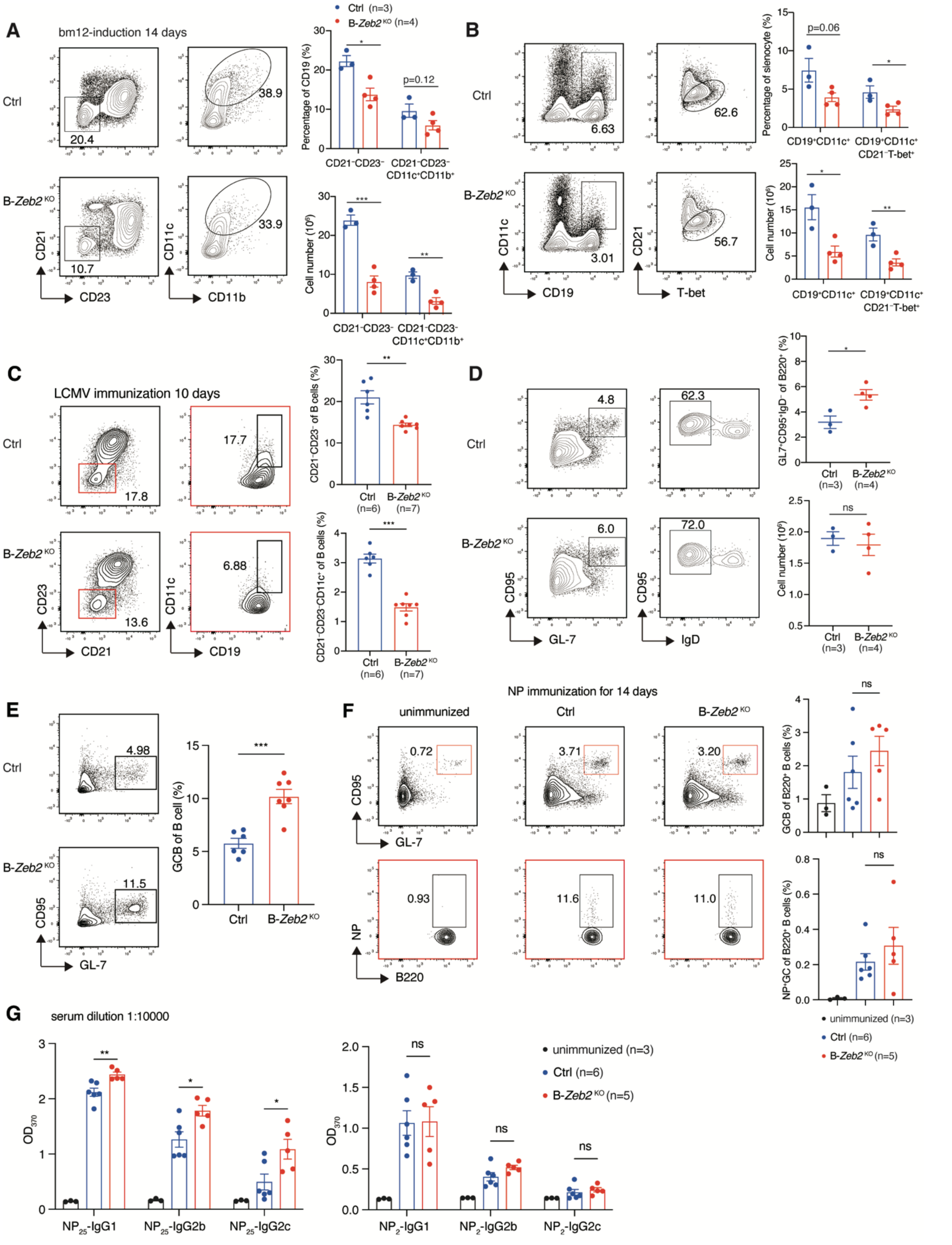


**Fig. S7. The effector B cell phenotype in different immunization models.** (**A** and **B**) Flow cytometry plots and the percentage and numbers of splenic ABCs ($CD21^{-}CD23^{-}$ B cells,

$CD21^-CD23^-CD11c^+CD11b^+$ B cells (A) and $CD19^+CD11c^+$, $CD19^+CD11c^+CD21^-$T-$bet^+$ (B) from B-*Zeb2*$^{KO}$ mice and control mice with bm12-induction of lupus. (**C**) The ABC phenotype in LCMV immunization model. (**D** and **E**) The GC B phenotype in bm12-induced (D) and LCMV immunization model (E). (**F**) Representative flow cytometry plots and frequency of GC B cells and NP-specific GC B cells in response to NP-CGG immunization. (**G**) $NP_{25}$-specific and $NP_2$-specific IgG1, IgG2b, and IgG2c antibody titers in the serum from B-*Zeb2*$^{KO}$ mice and control mice. n refers to the measurement of distinct samples (biological repeats). Data are representative of two independent experiments. Bars indicate mean ± SEM values. Statistical significance was analyzed using unpaired Student's *t* test (A to G and D for frequency of $CD21^-CD23^-CD11c^+$ B cells) and Welch's correction (D for frequency of $CD21^-CD23^-$ B cells). *$P<0.05$, **$P<0.01$, ***$P<0.001$, ns, not significant.

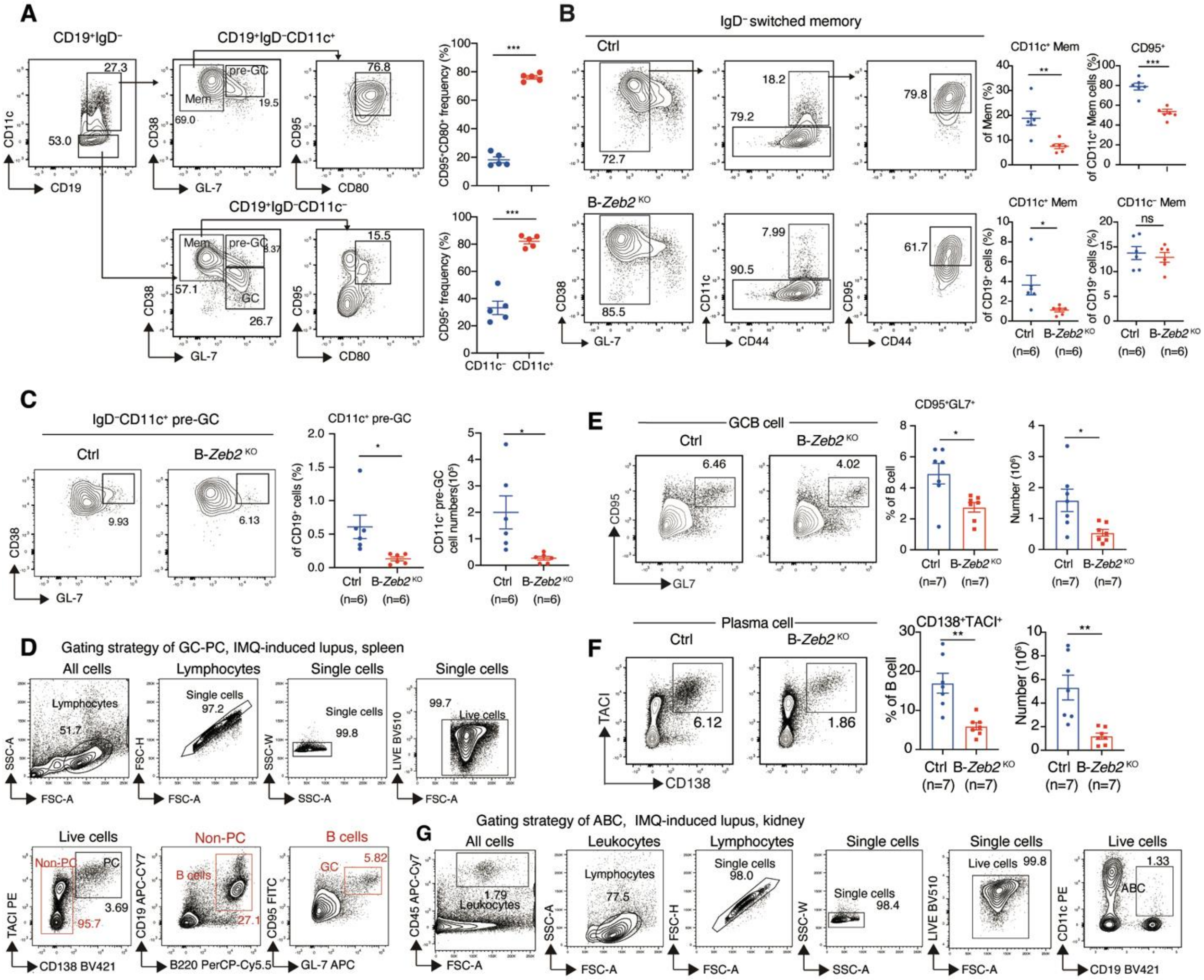


**Fig. S8. The effector B cell phenotype of B cell–conditional *Zeb2*-deficient mice after IMQ-induced lupus.** (**A**) Gating strategy (right) and frequency (left) of activated memory B cell fraction ($CD95^{+}CD38^{hi}GL\text{-}7^{-}$) in $IgD^{-}CD19^{hi}CD11c^{+}$ and $IgD^{-}CD19^{hi}CD11c^{-}$ B cells in IMQ-induced lupus mice. (**B**) Gating strategy (right) and frequency (left) of $CD11c^{+}$ memory B cells, $CD11c^{-}$ memory B cells, $CD44^{hi}CD95^{+}CD11c^{+}$ memory B cells in IMQ-induced lupus mice. (**C**) Flow cytometry plots and frequency of $CD11c^{+}$ pre-GC-like B cells ($CD19^{+}IgD^{-}CD11c^{+}CD38^{+}$ $GL\text{-}7^{+}$) in IMQ-induced lupus mice. (**D** to **F**) Gating strategy of splenic GC B cells and plasma cells (D). Flow cytometry plots (left) and frequency (middle) and number (right) of the splenic GC B cells (E) and plasma cells (F) in IMQ-induced lupus mice**. (G)** Gating strategy of ABCs from kidney for Fig. 3J. n refers to the measurement of distinct samples (biological repeats). Data are representative of two independent experiments. Bars indicate mean ± SEM values. Statistical significance was analyzed using unpaired Student's *t* test (A, B, and E for frequency) with Welch's correction (C, E for number, and F). *$P$<0.05, **$P$<0.01, ***$P$<0.001, ns, not significant.

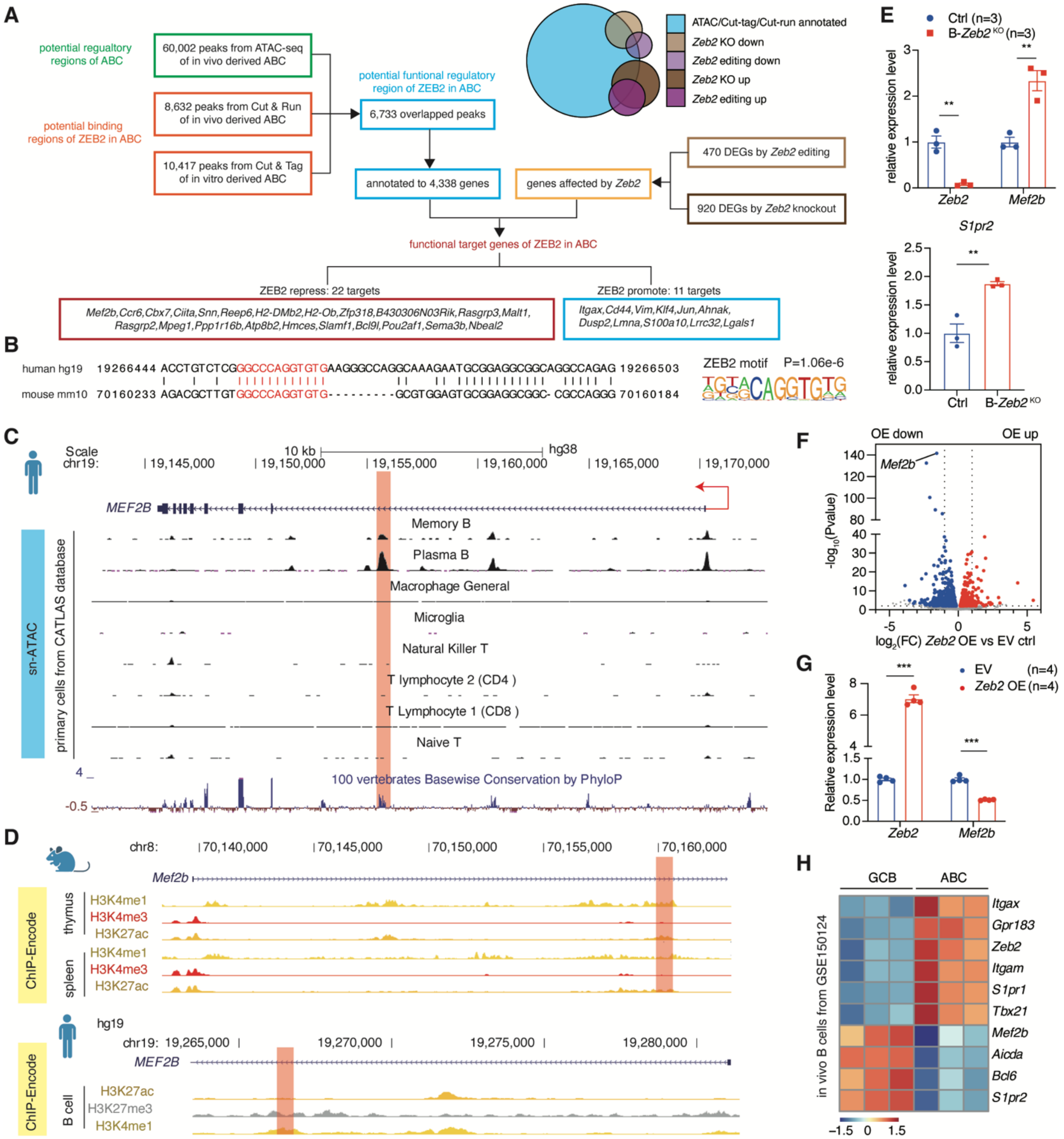


**Fig. S9. ZEB2 directly binds to the intronic enhancer of *Mef2b* to interfere with its expression.** (**A**) An analysis strategy diagram for the screening and identification of functional ZEB2 target genes in ABCs. The chromatin accessibility of ABCs was compared with two transcription factor binding site characterization approaches to reveal ZEB2 binding sites: CUT&Tag and CUT&RUN. After overlapping, 6733 accessible sites with ZEB2 binding were identified and annotated to 4338 genes. Among the genes differentially expressed by *Zeb2* deficiency, 33 candidate target genes of ZEB2 were identified, with 22 genes repressed and 11 genes activated by ZEB2. (**B**) The conserved sequence of *Mef2b +20 kb* intronic enhancer region with a ZEB2 binding motif. (**C**) The chromatin accessibility around *MEF2B* locus in human primary immune cell subsets from single nuclear ATAC data (CATLAS database) mapped in hg38. (**D**) ChIP-seq tracks display histone

modifications around the *MEF2B* locus in human (hg19) and the *Mef2b* locus in mouse (mm10) from Encode database. (**E**) Real-time PCR detection of *Zeb2*, *Mef2b*, and *S1pr2* expression between *Zeb2*-deficient B cells and control B cells. (**F**) Volcano graph showing the transcriptional profiles in *Zeb2*-overexpressing B cells. (**G**) Real-time PCR detection of *Zeb2* and *Mef2b* expression in *Zeb2*-overexpressing B cells. (**H**) The heatmap shows the expression of the selected genes between ABC and GC B cells from GSE150124. n refers to the measurement of distinct samples (biological repeats). Data are representative of two independent experiments (E). Bars indicate mean ± SEM values. Statistical significance was analyzed using unpaired Student's *t* test (E and G). *$P<0.05$, **$P<0.01$, ***$P<0.001$, ns, not significant.

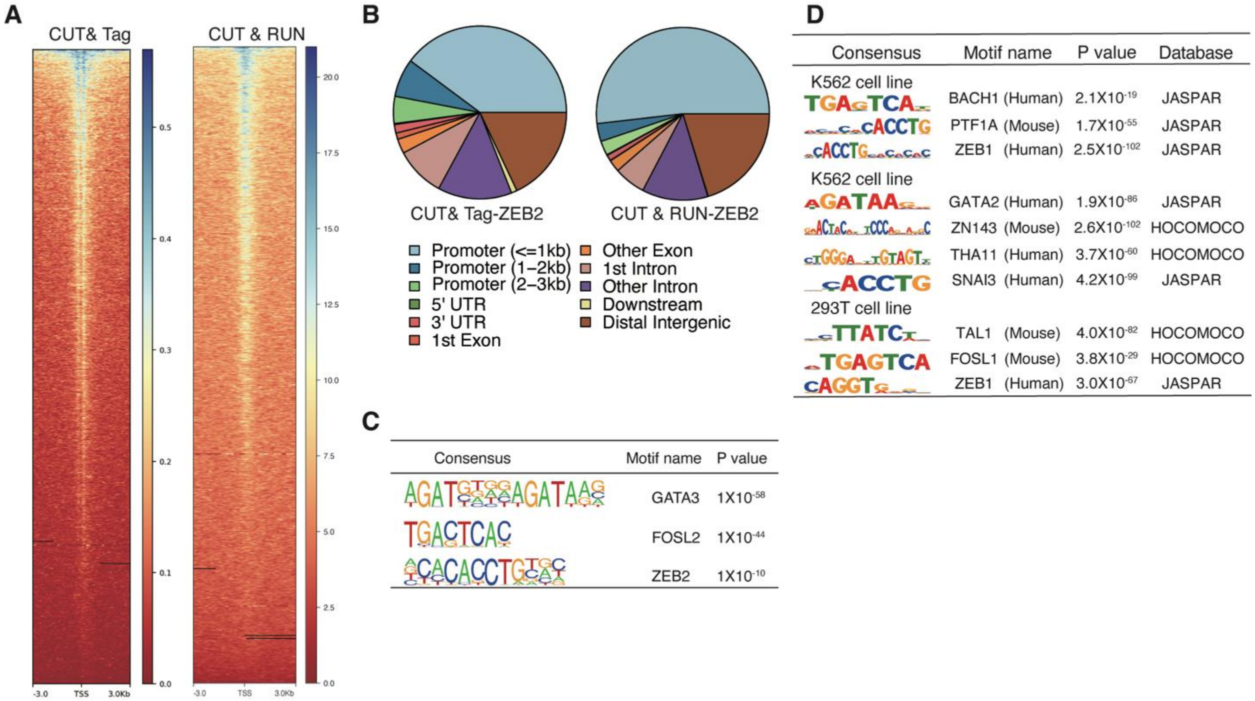


**Fig. S10. The transcriptional regulation of ZEB2 in mouse ABC analyzed by CUT&Tag and CUT&RUN.** (**A**) Heatmap graph showing distributions of ZEB2-specific signaling from CUT&Tag and CUT&RUN. The horizontal axis represented the normalized gene range coordinates. (**B**) Peak annotation analysis of distribution of ZEB2-specific peak in the functional area of gene covered. (**C**) Motif-enrichment analysis of ZEB2-specific peaks generated from CUT&Tag sequencing data of in vitro–derived ABCs. (**D**) Motif analysis of ZEB2-specific peak of ChIP-seq data in cell lines from Encode project.

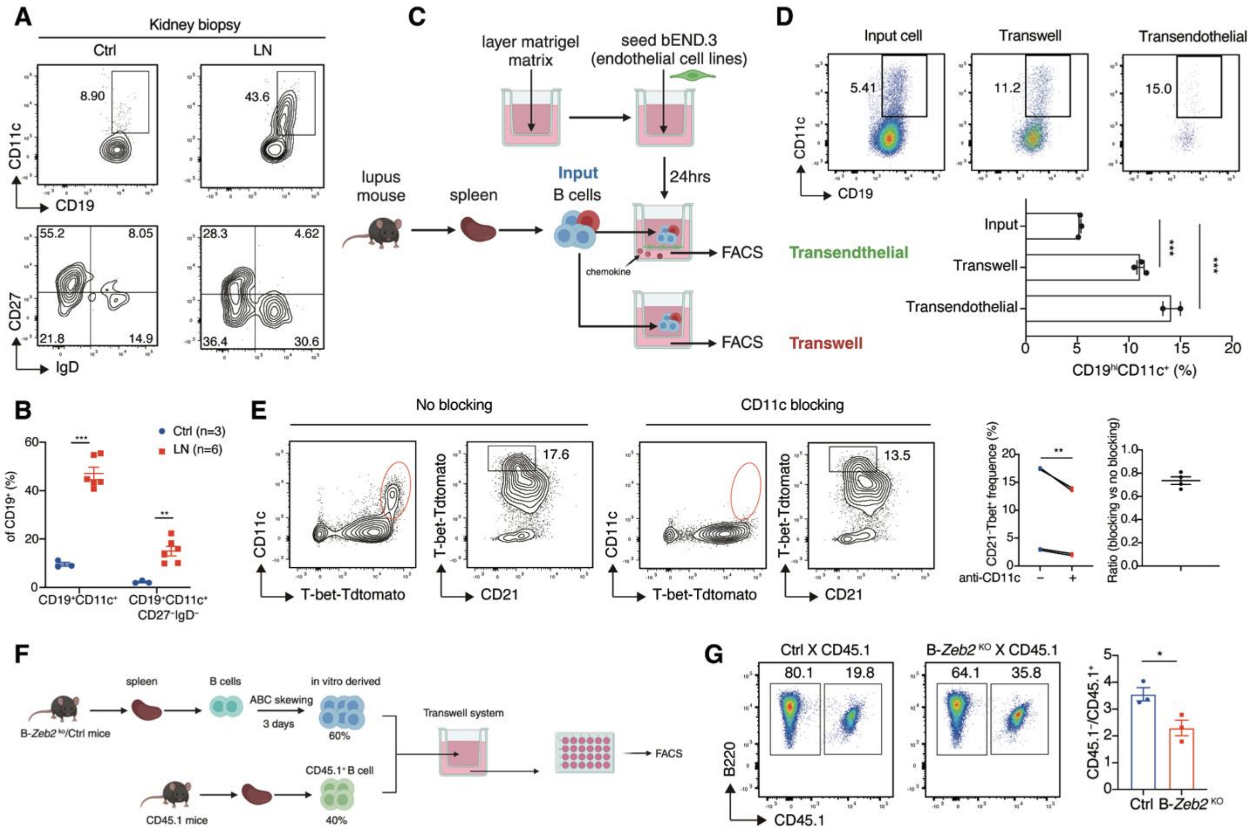


**Fig. S11. ABCs acquire enhanced migratory capacity via upregulated expression of the beta2 integrin CD11c controlled by ZEB2. (A and B)** Representative plots (A) and frequency (B) of renal CD11c$^+$ B cells and ABC (CD19$^+$CD11c$^+$CD27$^-$IgD$^-$) from paracancer control (Ctrl) and lupus nephritis (LN). (**C**) A diagram of the transwell and transendothelial migration assays. (**D**) Representative plots and frequency of the CD19$^{hi}$CD11c$^+$ among Input control, Transwell, and Transendothelial groups. (**E**) Representative plots and frequency of migrated ABCs with or without CD11c blocking. (**F**) Flow chart of migration assay for in vitro–induced ABCs. (**G**) Migratory capacity of in vitro–induced ABCs. n refers to the measurement of distinct samples (biological repeats). Data are representative of two independent experiments. Bars indicate mean ± SEM values. Statistical significance was analyzed using unpaired Student's *t* test (B, D, and G) and paired Student's *t* test (E). *$P$<0.05, ** $P$<0.01, ***$P$<0.001, ns, not significant.

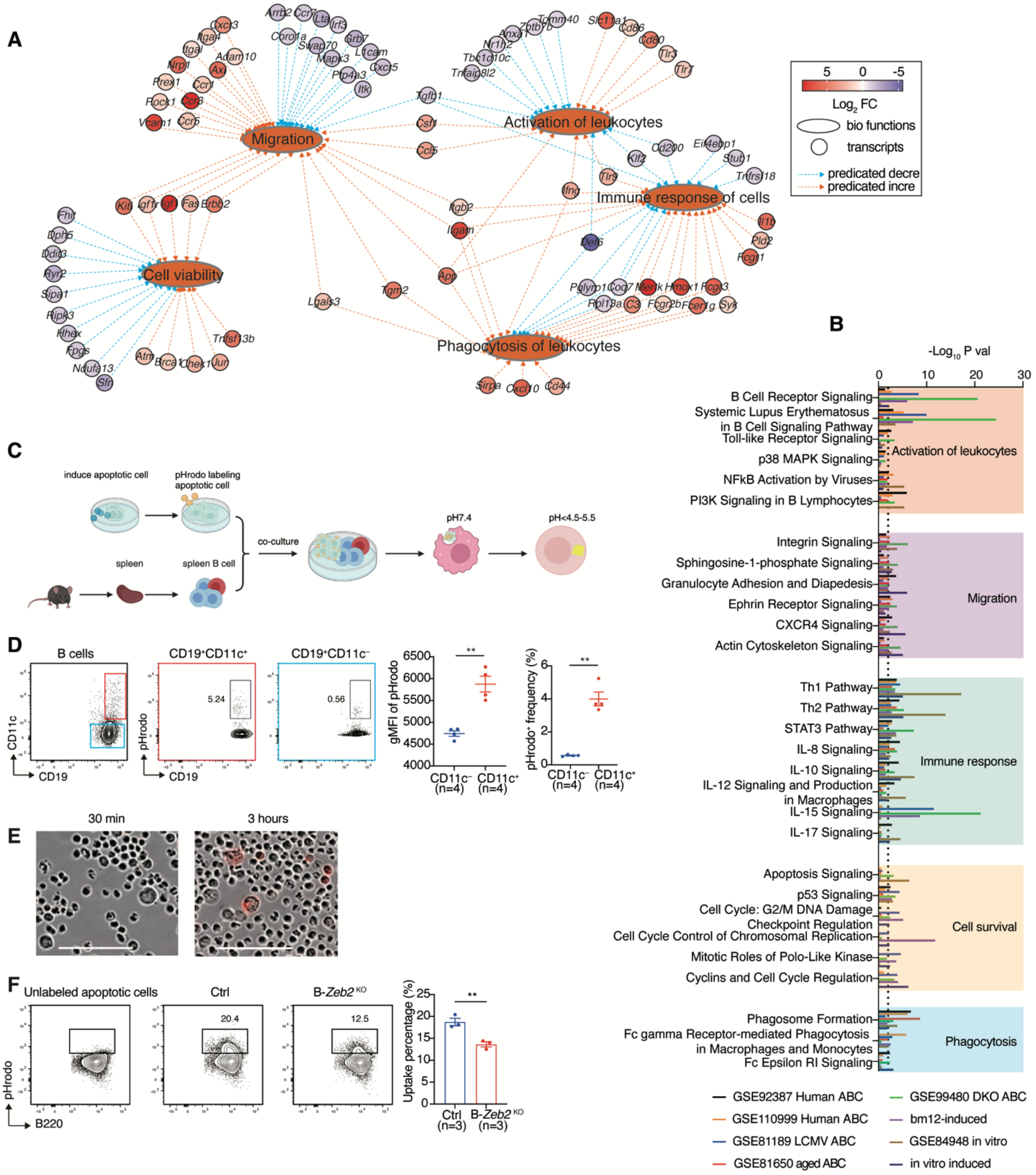


**Fig. S12. The distinct phagocytotic feature of ABCs is regulated by *Zeb2*.** (**A**) Biological functional classification and network analysis of differentially expressed genes (DEGs) using ingenuity pathways analysis (IPA). Dotted lines indicate the predicted relationship between genes and biological function, with orange representing activation and blue representing inhibition. (**B**) Pathway enrichment analysis of ABCs from datasets described in Fig. 4, B and G. The charts represent the top significantly signaling pathways related with functional categories: activation of leukocytes, migration, immune response, cell survival, and phagocytosis. (**C**) Flow chart of the in vitro phagocytosis assay. (**D**) Representative plots (left), uptake intensity (middle), and percentage

(right) of pHrodo-labeled apoptotic cells by splenic CD11c$^{+}$ and CD11c$^{-}$ B cells from IMQ-induced lupus mice. (**E**) Representative microscopy images of phagocytosis of pHrodo-labeled apoptotic cells by ABCs at indicated time points. Scale bars, 50 μm. (**F**) Representative plots (left) and uptake percentage (right) of the pHrodo-labeled apoptotic cells by in vitro–induced ABCs. n refers to the measurement of distinct samples (biological repeats). Data are representative of two independent experiments. Bars indicate mean ± SEM values. Statistical significance was analyzed using unpaired Student's *t* test (D for gMFI, and F) with Welch's correction (D for frequency). *$P$<0.05, **$P$<0.01, ***$P$<0.001, ns, not significant.

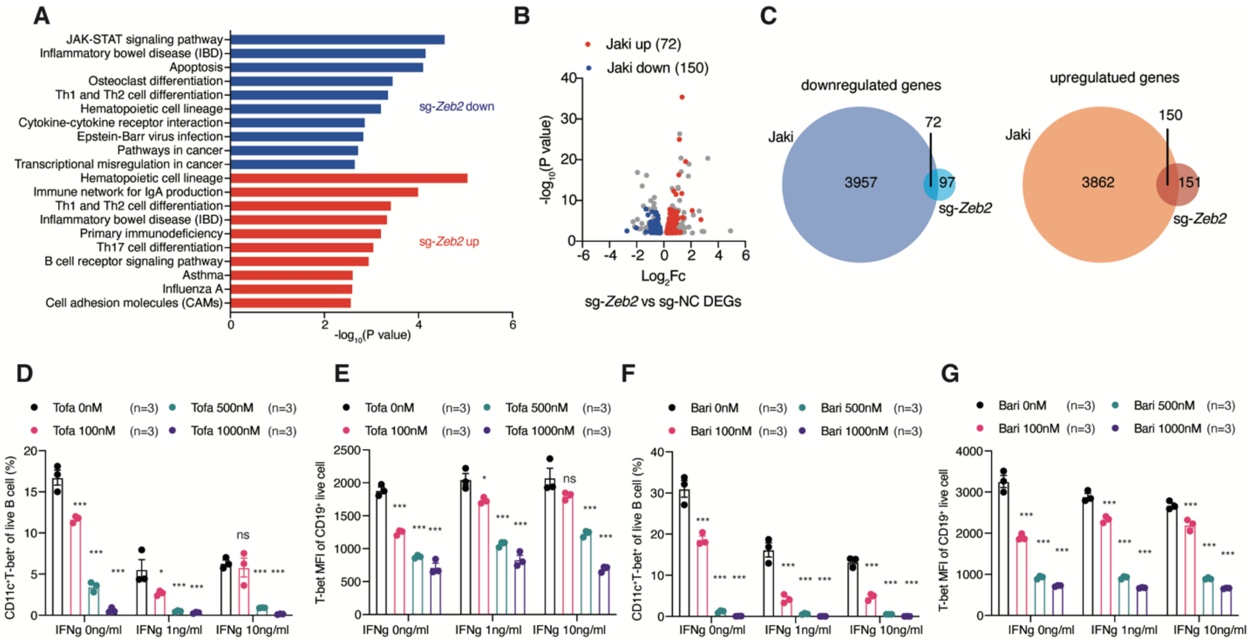

**Fig. S13. JAK–STAT inhibitors impair in vitro ABC differentiation.** (**A**) KEGG pathway enrichment analysis of downregulated (blue) and upregulated genes (red) in sg-*Zeb2* B cells. (**B** and **C**) Volcano plot (B) and Venn diagram (C) overlapping up- and downregulated genes by tofacitinib treatment with the affected genes by sg-*Zeb2* editing. (**D** and **E**) Frequency (D) and T-bet MFI (E) of in vitro–induced mouse CD11c$^+$T-bet$^+$ ABCs in addition of different concentrations of tofacitinib. (**F** and **G**) Frequency (F) and T-bet MFI (G) of in vitro–induced mouse CD11c$^+$T-bet$^+$ ABCs in addition of different concentrations of baricitinib (F). n refers to the measurement of distinct samples (biological repeats). Data are representative of two independent experiments. Bars indicate mean ± SEM values. Statistical analysis was performed using ordinary one-way ANOVA with two-sided Dunnett's multiple comparisons testing (D to G). **P*<0.05, ***P*<0.01, ****P*<0.001, ns, not significant.

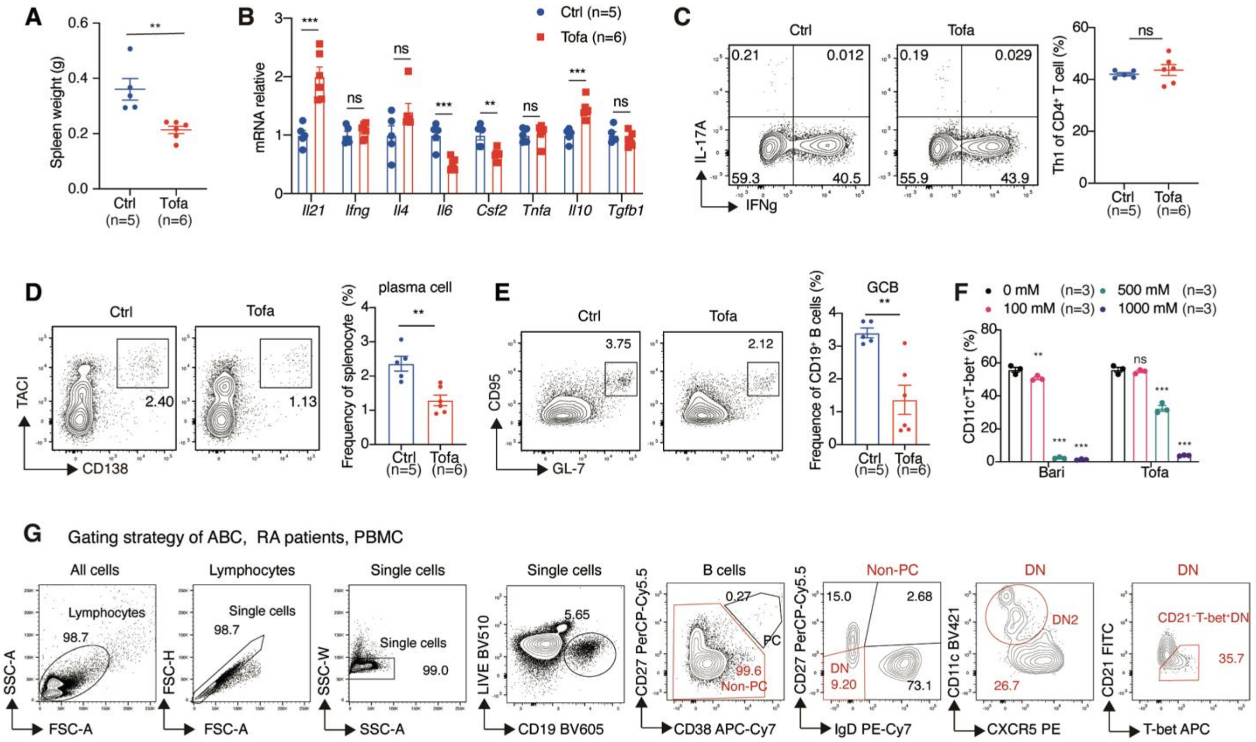


**Fig. S14. The effects of JAK–STAT inhibitors on bm12-induced lupus mice and RA patients.** (**A**) Spleen weight in bm12-indued lupus mice with tofacitinib treatment. (**B**) The expression of proinflammatory cytokines in splenocytes by qPCR. (**C** to **E**) Flow cytometry plots and frequency of the Th1 (C), plasma cells (D), and GC B cells (E) from tofacitinib-treated and control mice described in (A). (**F**) Frequency of in vitro–induced human ABCs in addition of different concentrations of tofacitinib or baricitinib. (**G**) Gating strategy of ABCs from PBMC of RA patients with tofacitinib treatment. Data are representative of two or three independent experiments (B to F). Bars indicate mean ± SEM values. Statistical significance was analyzed using unpaired Student's *t* test (A, B and D), Welch's correction (C and E) and ordinary one-way ANOVA with two-sided Dunnett's multiple comparisons testing (F). *$P$<0.05, **$P$<0.01, ***$P$<0.001, ns, not significant.

**Table S1. Basic clinical characteristics of a patient with new-onset SLE**

| | SLE patient |
|---|---|
| Age | 27 |
| Sex | Female |
| WBC (×10^9 cell/liter) | 4.35 |
| RBC (×10^12 cell/liter) | 3.14 |
| Hb (g/liter) | 106 |
| PLT (×10^9/liter) | 212 |
| ESR (mm/h) | 82 |
| IgA (g/liter) | 4.23 |
| IgM (g/liter) | 1.5 |
| IgG (g/liter) | 26.5 |
| C3 (g/liter) | 0.149 |
| C4 (g/liter) | <0.017 |
| Anti-dsDNA (IU/ml) | 292.16 |
| SLEDAI | 24 |
| ANA | Positive |
| Anti-SSA | Negative |
| Anti-SSB | Negative |
| Anti-U1RNP | Negative |
| Anti-Sm | Negative |
| Anti-rRNP | Positive |
| Anti-ANUA | Positive |
| ACL | Positive |
| Fever | No |
| Rash | Yes |
| Alpecia | Yes |
| Arthritis | No |
| Oral ulcers | No |
| Cutaneous vasculitis | No |
| Raynaud's | No |
| Leukopenia and/or Thrombocytopenia | No |
| Anemia | No |
| Serositis | No |
| NPSLE | No |
| LN | III+V |
| Urine protein (mg/24 hours) | 3379.8 |

**Table S2. Demographic and genetic information of MWS patients**

| Patient ID | Sex | Age(year) | Variants in ZEB2 | dbSNP ID | Inheritance |
|---|---|---|---|---|---|
| MWS-A1 | M | 5 | c.1027C>T p.Arg343X | rs786204815 | De novo |
| MWS-B1 | F | 2 | c.2851C>T p.Gln951X | - | De novo |
| MWS-C1 | M | 4 | c.1005delT p.Ile335Metfs*2 | - | De novo |
| MWS-D1 | F | 4 | chr2:138434153-145285163 del ZEB2 loss exon 1-10 | - | De novo |
| MWS-E1 | F | 1 | chr2:145147017-145274917 del ZEB2 loss exon 2-10 | - | De novo |

**Table S3. MWS patients clinical features**

| Patient ID | Symptoms | | | | | | Infectious disease | Autoimmune disease | |
|---|---|---|---|---|---|---|---|---|---|
| | Distinctive facial features | Developmental delay | Intellectual disability | Epilepsy | Hirschsprung disease | Congenital malformations | Upper respiratory tract infection (URTI) | ANA (1:80) | ENA[1] (1:100) |
| MWS-A1 | + | + | + | + | + | + | 4-5 times a year | - | - |
| MWS-B1 | + | + | + | + | - | + | 12 times a year | - | - |
| MWS-C1 | + | + | + | - | - | - | 3-4 times a year | - | - |
| MWS-D1 | + | + | + | + | - | - | 3 times a year | - | - |
| MWS-E1 | + | + | + | + | - | + | - | - | - |

+ feature present; - feature absent

[1] including anti-nRNP/Sm, anti-Sm, anti-SS-A, anti-Ro-52, anti-SS-B, anti-Scl-70, anti-Jo-1 and anti-Rib-P

## Table S4. Basic clinical characteristics of SLE patients with nephritis

| | LN biopsy(n=6) |
|---|---|
| Age (years), mean (SD) | 31.5 (5.32) |
| Female, n (%) | 6 (100) |
| SLEDAI | 10.5 (2.2) |
| rSLEDAI | 4 (0) |
| Urine protein (mg/24 hours) | 4.3 (3.0) |
| ESR (mm/h) | 33.0 (24.9) |
| IgG (g/liter) | 12.9 (9.1) |
| IgA (g/liter) | 2.9 (0.7) |
| IgM (g/liter) | 0.8 (0.4) |
| C3 (g/liter) | 0.4 (0.1) |
| C4 (g/liter) | 0.08 (0.05) |
| dsDNA (IU/ml) | 63.9 (30.6) |
| eGFR-EPI | 88.3 (18.6) |
| ANA | 1067 (330) |
| Renal pathology (ISN/RPS classification), n (%) | |
| II | 0 (0) |
| III | 0 (0) |
| IV | 3 (50) |
| V | 0 (0) |
| III+V | 0 (0) |
| IV+V | 3 (50) |

*SD:Standard deviation; n:number

## Table S5. Top 35 biological function analysis of DSE99480 DKO ABC vs WT FoB

| Representative Biological Functions | Biological Functions Annotation by IPA | P-value | Predicted Activation State | Activation z-score |
|---|---|---|---|---|
| Cell viability | Cell viability | 3.24E-24 | Increased | 6.955 |
| | Cell survival | 3.04E-25 | Increased | 6.793 |
| Migration of cells | Cell movement | 1.15E-33 | Increased | 6.462 |
| | Migration of cells | 4.42E-31 | Increased | 6.432 |
| | Homing of cells | 1.62E-16 | Increased | 5.406 |
| | Chemotaxis | 2.79E-16 | Increased | 5.174 |
| | Adhesion of blood cells | 1.48E-20 | Increased | 5.017 |
| | Adhesion of immune cells | 7.4E-20 | Increased | 4.729 |
| Phagocytosis of leukocytes | Phagocytosis | 6.71E-20 | Increased | 5.94 |
| | Internalization of cells | 3.95E-14 | Increased | 5.919 |
| | Phagocytosis of cells | 1.37E-19 | Increased | 5.878 |
| | Engulfment of cells | 7.49E-20 | Increased | 5.775 |
| | Engulfment of phagocytes | 2.64E-17 | Increased | 5.355 |
| | Engulfment of blood cells | 2.36E-14 | Increased | 5.333 |
| | Engulfment of myeloid cells | 9.02E-16 | Increased | 5.332 |
| | Engulfment of leukocytes | 2.6E-17 | Increased | 5.305 |
| | Binding of blood cells | 2.07E-20 | Increased | 5.249 |
| | Endocytosis | 3.33E-18 | Increased | 5.245 |
| | Endocytosis by eukaryotic cells | 2.98E-15 | Increased | 5.225 |
| | Engulfment by macrophages | 4.51E-15 | Increased | 5.14 |
| | Engulfment of antigen presenting cells | 5.29E-15 | Increased | 5.015 |
| | Binding of leukocytes | 9.89E-21 | Increased | 5.01 |
| | Phagocytosis of myeloid cells | 1.86E-16 | Increased | 4.938 |
| | Phagocytosis of blood cells | 1.78E-13 | Increased | 4.857 |
| | Phagocytosis of phagocytes | 3.17E-16 | Increased | 4.845 |
| | Phagocytosis of leukocytes | 6.94E-17 | Increased | 4.819 |
| Activation of lymphocytes | Organization of cytoskeleton | 4.15E-32 | Increased | 5.733 |
| | Organization of cytoplasm | 9.07E-34 | Increased | 5.677 |
| | Microtubule dynamics | 8.08E-26 | Increased | 5.215 |
| | Activation of cells | 1.65E-34 | Increased | 4.779 |
| Immune response of cells | Immune response of cells | 5.3E-27 | Increased | 5.577 |
| | Response of myeloid cells | 2.18E-20 | Increased | 5 |
| | Response of phagocytes | 1.29E-21 | Increased | 4.854 |
| | Immune response of myeloid cells | 2.44E-17 | Increased | 4.809 |
| | Interaction of blood cells | 2.15E-20 | Increased | 5.328 |

## Table S6. Clinical characteristics of RA patients treated with tofacitinib

| RA patients | Before (n=6) | After (n=6) |
|---|---|---|
| WBC (×10^9 cell/liter) | 6.30±2.92 | 6.30±2.03 |
| RBC (×10^12 cell/liter) | 4.73±0.56 | 4.70±0.62 |
| Hb (g/liter) | 136±25.25 | 137±23.50 |
| PLT (×10^9/liter) | 322±127.5 | 274±120 |
| ESR (mm/h) | 28±25.88 | 19±11.00 |
| CRP (mg/liter) | 10.4±32.80 | 6.6±4.90 |
| Cr (µmol/liter) | 56.2±8.93 | 62.2±13.85 |
| ALT (U/liter) | 21±11.25 | 25±7.00 |

# Table S7. List of antibodies

| Antibodies | Fluorochrome | Source | Identifier | dilution (final conc.) |
|---|---|---|---|---|
| anti-mouse CD4 | BV421 | Biolegend | Clone GK1.5 | 1:200 (1 μg/ml) |
| anti-mouse CD4 | AF488 | Biolegend | Clone GK1.5 | 1:200 (2.5 μg/ml) |
| anti-mouse CD8a | PE-Cy7 | Biolegend | Clone 53-6.7 | 1:200 (1 μg/ml) |
| anti-mouse CD19 | BV421 | Biolegend | Clone 6D5 | 1:100 (2 μg/ml) |
| anti-mouse CD19 | APC | Biolegend | Clone 6D5 | 1:100 (2 μg/ml) |
| anti-mouse CD19 | APC-Cy7 | Biolegend | Clone 6D5 | 1:100 (2 μg/ml) |
| anti-mouse CD19 | BV711 | Biolegend | Clone 6D5 | 1:100 (2 μg/ml) |
| anti-mouse CD19 | PerCP-Cy5.5 | Biolegend | Clone 1D3/CD19 | 1:100 (2 μg/ml) |
| anti-mouse CD44 | PerCP-Cy5.5 | BD Bioscience | Clone IM7 | 1:200 (1 μg/ml) |
| anti-mouse CD62L | FITC | BD Bioscience | Clone MEL-14 | 1:200 (2.5 μg/ml) |
| anti-mouse PD-1 | APC | eBioscience | Clone J43 | 1:100 (2 μg/ml) |
| anti-mouse B220 | APC | Biolegend | Clone RA3-6B2 | 1:200 (1 μg/ml) |
| anti-mouse B220 | APC-Cy7 | Biolegend | Clone RA3-6B2 | 1:200 (1 μg/ml) |
| anti-mouse B220 | FITC | Biolegend | Clone RA3-6B2 | 1:200 (2.5 μg/ml) |
| anti-mouse CD95 | PE | BD Bioscience | Clone Jo2 | 1:100 (2 μg/ml) |
| anti-mouse CD95 | FITC | BD Bioscience | Clone Jo2 | 1:100 (5 μg/ml) |
| anti-mouse GL-7 | AF647 | Biolegend | Clone GL7 | 1:200 (2.5 μg/ml) |
| anti-mouse GL-7 | FITC | BD Bioscience | Clone GL7 | 1:100 (5 μg/ml) |
| anti-mouse IgM | FITC | BD Bioscience | Clone R6-60.2 | 1:200 (2.5 μg/ml) |
| anti-mouse IgM | BV510 | BD Bioscience | Clone R6-60.2 | 1:200 (1 μg/ml) |
| anti-mouse IgD | BV421 | Biolegend | Clone 11-26c.2a | 1:300 (0.66 μg/ml) |
| anti-mouse IgD | BV605 | Biolegend | Clone 11-26c.2a | 1:300 (0.66 μg/ml) |
| anti-mouse CD138 | BV421 | Biolegend | Clone 281-2 | 1:200 (1 μg/ml) |
| anti-mouse CD138 | PE | Biolegend | Clone 281-2 | 1:200 (1 μg/ml) |
| anti-mouse TACI | PE | Biolegend | Clone 8F10 | 1:100 (2 μg/ml) |
| anti-mouse CD11c | BV421 | Biolegend | Clone N418 | 1:100 (2 μg/ml) |
| anti-mouse CD11c | PE | Biolegend | Clone N418 | 1:100 (2 μg/ml) |
| anti-mouse/human T-bet | APC | Biolegend | Clone 4B10 | 1:100 (2 μg/ml) |
| anti-mouse CD21 | FITC | Biolegend | Clone 7E9 | 1:200 (2.5 μg/ml) |
| anti-mouse CD21 | APC-Cy7 | Biolegend | Clone 7E9 | 1:200 (1 μg/ml) |
| anti-mouse CD73 | BV605 | Biolegend | Clone TY/11.8 | 1:100 (1 μg/ml) |
| anti-mouse CD80 | PE-Cy7 | Biolegend | Clone 16-10A1 | 1:100 (2 μg/ml) |
| anti-mouse CD93 | PE | BD Bioscience | Clone AA4.1 | 1:100 (2 μg/ml) |
| anti-mouse CD45.1 | PE-Cy7 | Biolegend | Clone A20 | 1:100 (2 μg/ml) |
| anti-mouse CD86 | BV605 | Biolegend | Clone GL1 | 1:200 (0.5 μg/ml) |
| anti-mouse CD86 | PE-Cy7 | BD Bioscience | Clone GL1 | 1:200 (1 μg/ml) |
| anti-mouse CD86 | APC | BD Bioscience | Clone GL1 | 1:200 (1 μg/ml) |
| anti-mouse I-A/I-E | FITC | Biolegend | Clone M5/114.15.2 | 1:200 (2.5 μg/ml) |
| anti-mouse I-A/I-E | BV421 | BD Bioscience | Clone M5/114.15.2 | 1:200 (1 μg/ml) |
| anti-mouse CD23 | PE-Cy7 | BD Bioscience | Clone B3B4 | 1:100 (2 μg/ml) |
| anti-mouse CD11b | PerCP-Cy5.5 | Biolegend | Clone M1/70 | 1:100 (2 μg/ml) |
| anti-mouse CD5 | APC | eBioscience | Clone 53-7.3 | 1:100 (2 μg/ml) |
| anti-mouse CXCR4 | PE | BD Bioscience | Clone 2B11/CXCR4 | 1:100 (2 μg/ml) |
| anti-mouse CXCR3 | APC | Biolegend | Clone CXCR3-173 | 1:100 (2 μg/ml) |
| anti-human CD19 | BV605 | Biolegend | Clone HIB19 | 1:100 (0.8 μg/ml) |
| anti-human CD19 | BV650 | Biolegend | Clone HIB19 | 1:200 |
| anti-human CXCR5 | PE | Biolegend | Clone J252D4 | 1:100 (1 μg/ml) |
| anti-human CXCR5 | AF647 | BD Bioscience | Clone RF8B2 | 1:200 |
| anti-human IgD | PE-Cy7 | Biolegend | Clone IA6-2 | 1:300 (0.13 μg/ml) |
| anti-human IgD | BV510 | Biolegend | Clone IA6-2 | 1:300 |
| anti-human CD27 | PerCP-Cy5.5 | Biolegend | Clone M-T271 | 1:100 (2 μg/ml) |
| anti-human CD11c | BV421 | Biolegend | Clone S-HCL-3 | 1:100 (1.2 μg/ml) |
| anti-human CD11c | BUV395 | BD Bioscience | Clone B-ly6 | 1:200 |
| anti-human CD38 | PE | Biolegend | Clone HIT2 | 1:200 (0.5 μg/ml) |
| anti-human CD38 | BV605 | Biolegend | Clone HIT2 | 1:200 |
| anti-human CD21 | FITC | Biolegend | Clone Bu32 | 1:200 (0.5 μg/ml) |
| anti-human CD3 | BV786 | BD Bioscience | Clone SK7 | 1:200 |
| anti-human CD4 | BUV496 | BD Bioscience | Clone SK3 | 1:200 |
| anti-human CD56 | BUV737 | BD Bioscience | Clone NCAM16.2 | 1:200 |
| anti-human CD45RA | PE-Cy7 | eBioscience | Clone HI100 | 1:200 |
| Biotin Rat Anti-Mouse CD185 (CXCR5) | biotin | BD Bioscience | Clone 2G8 | 1:100 (5 μg/ml) |
| Zombie Aqua™ Fixable Viability Kit | --- | Biolegend | Cat#423102 | 1:1000 |
| PE Streptavidin | --- | BD Bioscience | Cat#554061 | 1:300 (1.66 μg/ml) |
| T-bet (4B10) Antibody | --- | Santa Cruz Biotechnology | Cat#sc-21749 | 1:100 (2 μg/ml) |
| ZEB2 antibody | --- | Novus | Cat#NBP1-82991 | 1:50 (2 μg/ml) |

# Table S8. List of sgRNA sequence

| Gene name | Sg sequence(5′-3′) | ICE indel (%) |
|---|---|---|
| mouse | | |
| *Zeb2 Sg1* | GTACCTTCAGCGAAGCGACA | 57 |
| *Zeb2 Sg2* | TATGAATAGTAACTTGAGTG | 68 |
| *Tbx21* | CGAGGACTACGCATTGCCCG | 84 |
| *Itgax* | GGGCCGTAACTCACCCTGGA | 69 |
| *Zbtb32* | CGAGGTATCGAGAGCCACAA | 81 |
| *Tfeb* | CCTCTGTGGATTACATCCGG | 57 |
| *Litaf* | GATAACAGACATACTTGCGT | 78 |
| *Nfatc2* | TTGGAGAGTGGCCACTCGAG | 76 |
| *Srebf2* | ACTCCAGTGACAGTACACTG | 72 |
| *Jazf1* | CACAGGCAGCGAGTATGATG | 53 |
| *Jun* | TGTGCCGCGGAGGTGACACT | 84 |
| *Plek* | GTTTGCCAAAGTCTTGACAA | 46 |
| *Batf* | AGAGATCAAACAGCTCACCG | 84 |
| *Tfec* | CATCAGTGGACTACATCAAG | 69 |
| *Sox5* | CGAGGGTCCGCTGGTCAGGA | 74 |
| *Mbd4* | TCAGAGTCGCCAGAAAGCAG | 90 |
| *Ets1* | TGCCTGGGGAGAGCCAGTCG | 60 |
| *Fli1* | TCACGACTGAATGTCAAGGA | 66 |
| human | | |
| *ZEB2 Sg1* | TTGTAGCCCCGGTCGCAGTA | 56 |
| *ZEB2 Sg2* | GGCGCAAACAAGCCAATCCC | 50 |
| *TBX21* | AAACCGCCTGTACGTCCACC | 62 |
| *BATF* | AGGACTCTACCTGTTTGCCA | 63 |
| *FLI1* | ACTCAATCGTGAGGATTGGT | 77 |
| *SREBF2* | GCTGCATTCTGGTATATCAA | 64 |
| *ETS1* | CTTACTAATGAAGTAATCCG | 46 |
| *JUN* | TGAACCTGGCCGACCCAGTG | 33 |
| *CD19* | CTGTGCTGCAGTGCCTCAA | |

## Table S9. List of primers

| | Gene name | Forward | Reverse |
|---|---|---|---|
| Mouse | | | |
| | *Hopx* | 5′-CAACTTCAACAAGGTCAACAAGCAC-3′ | 5′-ACCATTTCTGCGTCTGCTCCT-3′ |
| | *Zeb2* | 5′-GCAGTGAGCATCGAAGAGTACC-3′ | 5′-GGCAAAAGCATCTGGAGTTCCAG-3′ |
| | *Tbx21* | 5′-TGTGGATGTGGTCTTGGTGG-3′ | 5′-ATTGTTGGAAGCCCCCTTGT-3′ |
| | *Itgax* | 5′-TTGGCTTGTGGTCCTACTGTG-3′ | 5′-GGGAAGTTCTGGCTCTGCTTG-3′ |
| | *Itgam* | 5′-GTGAATATGTCCTTGGGCCTGT -3′ | 5′-CGGAGCCATCAATCAAGAAGACA -3′ |
| | *Zbtb32* | 5′-TCCAGATACGGTGCTCCCTTCT-3′ | 5′-CCAGAGAGCTTTGGAGTGGTTC-3′ |
| | *Cxcl10* | 5′-ATCATCCCTGCGAGCCTATCCT-3′ | 5′-GACCTTTTTTGGCTAAACGCTTTC-3′ |
| | *Rpl13a* | 5′-GGGCAGGTTCTGGTATTGGAT-3′ | 5′-GGCTCGGAAATGGTAGGGG-3′ |
| | *Cxcl9* | 5′-AATGCACGATGCTCCTGCA-3′ | 5′-AGGTCTTTGCGGGATTTGTAGTGG-3′ |
| | *Bach2* | 5′-ACAGACGAAAGATGACTTGGTG-3′ | 5′-CTCTGCTGAGTAACAGCTTGG-3′ |
| | *Myc* | 5′-ACGGCCTTCTCTCCTTCCTC-3′ | 5′-GCCTCTTCTCCACAGACACC-3′ |
| | *Nfatc2* | 5′-TCATCCAACAACAGACTGCCC-3′ | 5′-GGGAGGGAGGTCCTGAAAACT-3′ |
| | *Junb* | 5′-TCACGACGACTCTTACGCAG-3′ | 5′-CCTTGAGACCCCGATAGGGA-3′ |
| | *Jun* | 5′-CCTTCTACGACGATGCCCTC-3′ | 5′-GGTTCAAGGTCATGCTCTGTTT-3′ |
| | *Srebf2* | 5′-AGAAAGAGCGGTGGAGTCCTTG-3′ | 5′-GAACTGCTGGAGAATGGTGAGG-3′ |
| | *Foxp1* | 5′-CATGCCTCTACCAATGGACAGC-3′ | 5′-GAAGTCGTCACAAACCGCCTCA-3′ |
| | *Zfp36l1* | 5′-CTTCCACACCATCGGCTTTTGC-3′ | 5′-CACTGGGAAACCCAGCAAAGCT-3′ |
| | *Aff3* | 5′-AGATGACCTGGCTTCCTCCACT-3′ | 5′-GTCCAGAGGATACAAGTTGCGAG-3′ |
| | *Tfeb* | 5′-CCACCCCAGCCATCAACAC-3′ | 5′-CAGACAGATACTCCCGAACCTT-3′ |
| | *Klf6* | 5′-GGAAGGTTGTGAGTGGCGTTTTG-3′ | 5′-AGGTGGTCAGACCTGGAGAAAC-3′ |
| | *Irf1* | 5′-TCCAAGTCCAGCCGAGACACTA-3′ | 5′-ACTGCTGTGGTCATCAGGTAGG-3′ |
| | *Irf8* | 5′-CAATCAGGAGGTGGATGCTTCC-3′ | 5′-GTTCAGAGCACAGCGTAACCTC-3′ |
| | *Pou2f2* | 5′-TCCTGGAGAAGTGGCTCAACGA-3′ | 5′-ATGCTGGTCCTCTTCTTGCGTC-3′ |
| | *Litaf* | 5′-CAAGATGATCGTGACCCAGCTG-3′ | 5′-GCAGTAGTGGTCCACATCCTGT-3′ |
| | *Klf3* | 5′-CCTCTCATGGTTTCCTTGTCGG-3′ | 5′-CCTCTGTGGTTCAATTCCAGGC-3′ |
| | *Jazf1* | 5′-CGACATAGCAGTGGCAGCCTTA-3′ | 5′-TCTCTGTGGTCCAGGACTCATC-3′ |
| | *Plek* | 5′-GGAGCAGTTCACTTGAGAGGCT-3′ | 5′-TGGAAGTGGCTGCCTGCAAGTA-3′ |
| | *Plscr1* | 5′-GACCTCTGAGATGCAGTAGCTG-3′ | 5′-GGAGAGTGAACTTGGGCAGACA-3′ |
| | *Zfp36l2* | 5′-AAGTACGGCGAGAAGTGCCAGT-3′ | 5′-AGAAGCCGATGGTGTGGAAGGT-3′ |
| | *Fos* | 5′-AGGGGCAAAGTAGAGCAGCTA-3′ | 5′-CAATCTCAGTCTGCAACGCA-3′ |
| | *Tox* | 5′-AAGATGGCGCACTGCTCTCCAA-3′ | 5′-CATGCTTGCCTGCTGTCTGATG-3′ |
| | *Bhlhe40* | 5′-CGGATTAACGAGTGCATTGCCC-3′ | 5′-GCTTCAACGTAAGCTCCAGAACC-3′ |
| | *Foxp4* | 5′-CACCAGGATGTTCGCCTACTTC-3′ | 5′-TATTCCCGCTCATCCACAGTCC-3′ |
| | *Sox5* | 5′-CGCCAGATGAAAGAGCAACTCAG-3′ | 5′-TGAGTCAGGCTCTCCAGTGTTG-3′ |
| | *Crem* | 5′-GCAGCACAATCAGCCGATGGTA-3′ | 5′-AGCTCGGATCTGGTAAGTTGGC-3′ |
| | *Arid5a* | 5′-CGCCTCTGGAAGAACGTGTATG-3′ | 5′-TGGTAGGAGGCAGTGGCTTGTC-3′ |
| | *Arid3a* | 5′-TCCATCACCAGTGCTGCCTTCA-3′ | 5′-TCCCTGCGATTGCTGTCTATGG-3′ |
| | *Fli1* | 5′-CCATACAGACCAGTCCTCACGA-3′ | 5′-CATGGTCTGTGATCCTCCAAGG-3′ |
| | *Mbd4* | 5′-ACAGGATGGCTCTGAAATGCCC-3′ | 5′-ACTTGTGTCCGTGGGATGCTGT-3′ |
| | *Ets1* | 5′-CCAGAATCCTGTTACACCTCGG-3′ | 5′-CAGCGTCTGATAGGACTCTGTG-3′ |
| | *Hif1a* | 5′-CCTGCACTGAATCAAGAGGTTGC-3′ | 5′-CCATCAGAAGGACTTGCTGGCT-3′ |
| | *Mef2b* | 5′-AAGGTCCTGGAGAGAAGCTGCT-3′ | 5′-GTAGGACAGCTCTGAAACCGAC-3′ |
| | *S1pr2* | 5′-CTCACTGCTCAATCCTGTCATC-3′ | 5′-TTCACATTTTCCCTTCAGACC-3′ |
| | *Il21* | 5′-GATCCTGAACTTCTATCAGCTCCAC-3′ | 5′-GGCATTTAGCTATGTGCTTCTGTT-3′ |
| | *Ifng* | 5′-ATGAACGCTACACACTGCATC-3′ | 5′-CCATCCTTTTGCCAGTTCCTC-3′ |
| | *Il4* | 5′-ACTTGAGAGAGATCATCGGCA-3′ | 5′-AGCTCCATGAGAACACTAGAGTT-3′ |
| | *Il6* | 5′-GATGGATGCTACCAAACTGGAT-3′ | 5′-CCAGGTAGCTATGGTACTCCAGA-3′ |
| | *Csf2* | 5′-GTCTCTAACGAGTTCTCCTTCA-3′ | 5′-TAGTAGCTGGCTGTCATGTTC-3′ |
| | *Tnfa* | 5′-TCTTCTCATTCCTGCTTGTGG-3′ | 5′-GGTCTGGGCCATAGAACTGA-3′ |
| | *Il10* | 5′-TGAGAAGCTGAAGACCCTCA-3′ | 5′-ACCTTGGTCTTGGAGCTTATT-3′ |
| | *Tgfb1* | 5′-TGGAGCAACATGTGGAACTC-3′ | 5′-CAGCAGCCGGTTACCAAG-3′ |
| Human | | | |
| | *ZEB2* | 5′-AATGCACAGAGTGTGGCAAGGC-3′ | 5′-CTGCTGATGTGCGAACTGTAGG-3′ |
| | *RPL13A* | 5′-CGAGGTTGGCTGGAAGTACC-3′ | 5′-CTTCTCGGCCTGTTTCCGTAG-3′ |

## References and Notes


1. M. P. Cancro, Age-Associated B Cells. *Annu. Rev. Immunol.* **38**, 315–340 (2020).

2. Y. Hao, P. O'Neill, M. S. Naradikian, J. L. Scholz, M. P. Cancro, A B-cell subset uniquely responsive to innate stimuli accumulates in aged mice. *Blood* **118**, 1294–1304 (2011).

3. A. V. Rubtsov, K. Rubtsova, A. Fischer, R. T. Meehan, J. Z. Gillis, J. W. Kappler, P. Marrack, Toll-like receptor 7 (TLR7)-driven accumulation of a novel CD11c$^{+}$ B-cell population is important for the development of autoimmunity. *Blood* **118**, 1305–1315 (2011).

4. S. Wang, J. Wang, V. Kumar, J. L. Karnell, B. Naiman, P. S. Gross, S. Rahman, K. Zerrouki, R. Hanna, C. Morehouse, N. Holoweckyj, H. Liu, Autoimmunity Molecular Medicine Team, Z. Manna, R. Goldbach-Mansky, S. Hasni, R. Siegel, M. Sanjuan, K. Streicher, M. P. Cancro, R. Kolbeck, R. Ettinger, IL-21 drives expansion and plasma cell differentiation of autoreactive CD11c$^{hi}$T-bet$^{+}$ B cells in SLE. *Nat. Commun.* **9**, 1758 (2018).

5. S. A. Jenks, K. S. Cashman, E. Zumaquero, U. M. Marigorta, A. V. Patel, X. Wang, D. Tomar, M. C. Woodruff, Z. Simon, R. Bugrovsky, E. L. Blalock, C. D. Scharer, C. M. Tipton, C. Wei, S. S. Lim, M. Petri, T. B. Niewold, J. H. Anolik, G. Gibson, F. E.-H. Lee, J. M. Boss, F. E. Lund, I. Sanz, Distinct Effector B Cells Induced by Unregulated Toll-like Receptor 7 Contribute to Pathogenic Responses in Systemic Lupus Erythematosus. *Immunity* **49**, 725–739.e6 (2018).

6. E. Zumaquero, S. L. Stone, C. D. Scharer, S. A. Jenks, A. Nellore, B. Mousseau, A. Rosal-Vela, D. Botta, J. E. Bradley, W. Wojciechowski, T. Ptacek, M. I. Danila, J. C. Edberg, S. L. Bridges Jr., R. P. Kimberly, W. W. Chatham, T. R. Schoeb, A. F. Rosenberg, J. M. Boss, I. Sanz, F. E. Lund, IFNγ induces epigenetic programming of human T-bet$^{hi}$ B cells and promotes TLR7/8 and IL-21 induced differentiation. *eLife* **8**, e41641 (2019).

7. S. L. Stone, J. N. Peel, C. D. Scharer, C. A. Risley, D. A. Chisolm, M. D. Schultz, B. Yu, A. Ballesteros-Tato, W. Wojciechowski, B. Mousseau, R. S. Misra, A. Hanidu, H. Jiang, Z. Qi, J. M. Boss, T. D. Randall, S. R. Brodeur, A. W. Goldrath, A. S. Weinmann, A. F. Rosenberg, F. E. Lund, T-bet Transcription Factor Promotes Antibody-Secreting Cell Differentiation by Limiting the Inflammatory Effects of IFN-γ on B Cells. *Immunity* **50**, 1172–1187.e7 (2019).

8. M. Manni, S. Gupta, E. Ricker, Y. Chinenov, S. H. Park, M. Shi, T. Pannellini, R. Jessberger, L. B. Ivashkiv, A. B. Pernis, Regulation of age-associated B cells by IRF5 in systemic autoimmunity. *Nat. Immunol.* **19**, 407–419 (2018).

9. E. Ricker, M. Manni, D. Flores-Castro, D. Jenkins, S. Gupta, J. Rivera-Correa, W. Meng, A. M. Rosenfeld, T. Pannellini, M. Bachu, Y. Chinenov, P. K. Sculco, R. Jessberger, E. T. L. Prak, A. B. Pernis, Altered function and differentiation of age-associated B cells contribute to the female bias in lupus mice. *Nat. Commun.* **12**, 4813 (2021).

10. S. W. Du, T. Arkatkar, H. M. Jacobs, D. J. Rawlings, S. W. Jackson, Generation of functional murine CD11c$^{+}$ age-associated B cells in the absence of B cell T-bet expression. *Eur. J. Immunol.* **49**, 170–178 (2019).

11. R. C. Levack, K. L. Newell, M. Popescu, B. Cabrera-Martinez, G. M. Winslow, CD11c$^{+}$ T-bet$^{+}$ B Cells Require IL-21 and IFN-γ from Type 1 T Follicular Helper Cells and Intrinsic

Bcl-6 Expression but Develop Normally in the Absence of T-bet. *J. Immunol.* **205**, 1050–1058 (2020).

12. C. Lien, C.-M. Fang, D. Huso, F. Livak, R. Lu, P. M. Pitha, Critical role of IRF-5 in regulation of B-cell differentiation. *Proc. Natl. Acad. Sci. U.S.A.* **107**, 4664–4668 (2010).
13. H. Xu, V. K. Chaudhri, Z. Wu, K. Biliouris, K. Dienger-Stambaugh, Y. Rochman, H. Singh, Regulation of bifurcating B cell trajectories by mutual antagonism between transcription factors IRF4 and IRF8. *Nat. Immunol.* **16**, 1274–1281 (2015).
14. S. De, B. Zhang, T. Shih, S. Singh, A. Winkler, R. Donnelly, B. J. Barnes, B Cell-Intrinsic Role for IRF5 in TLR9/BCR-Induced Human B Cell Activation, Proliferation, and Plasmablast Differentiation. *Front. Immunol.* **8**, 1938 (2018).
15. D. R. Glass, A. G. Tsai, J. P. Oliveria, F. J. Hartmann, S. C. Kimmey, A. A. Calderon, L. Borges, M. C. Glass, L. E. Wagar, M. M. Davis, S. C. Bendall, An Integrated Multi-omic Single-Cell Atlas of Human B Cell Identity. *Immunity* **53**, 217–232.e5 (2020).
16. M. S. Naradikian, A. Myles, D. P. Beiting, K. J. Roberts, L. Dawson, R. S. Herati, B. Bengsch, S. L. Linderman, E. Stelekati, R. Spolski, E. J. Wherry, C. Hunter, S. E. Hensley, W. J. Leonard, M. P. Cancro, Cutting Edge: IL-4, IL-21, and IFN-γ Interact To Govern T-bet and CD11c Expression in TLR-Activated B Cells. *J. Immunol.* **197**, 1023–1028 (2016).
17. C. Vandewalle, J. Comijn, B. De Craene, P. Vermassen, E. Bruyneel, H. Andersen, E. Tulchinsky, F. Van Roy, G. Berx, SIP1/ZEB2 induces EMT by repressing genes of different epithelial cell-cell junctions. *Nucleic Acids Res.* **33**, 6566–6578 (2005).
18. D. R. Mowat, G. D. Croaker, D. T. Cass, B. A. Kerr, J. Chaitow, L. C. Adès, N. L. Chia, M. J. Wilson, Hirschsprung disease, microcephaly, mental retardation, and characteristic facial features: Delineation of a new syndrome and identification of a locus at chromosome 2q22-q23. *J. Med. Genet.* **35**, 617–623 (1998).
19. N. Wakamatsu, Y. Yamada, K. Yamada, T. Ono, N. Nomura, H. Taniguchi, H. Kitoh, N. Mutoh, T. Yamanaka, K. Mushiake, K. Kato, S. Sonta, M. Nagaya, Mutations in SIP1, encoding Smad interacting protein-1, cause a form of Hirschsprung disease. *Nat. Genet.* **27**, 369–370 (2001).
20. C. L. Scott, K. D. Omilusik, ZEBs: Novel Players in Immune Cell Development and Function. *Trends Immunol.* **40**, 431–446 (2019).
21. G. J. Brown, P. F. Cañete, H. Wang, A. Medhavy, J. Bones, J. A. Roco, Y. He, Y. Qin, J. Cappello, J. I. Ellyard, K. Bassett, Q. Shen, G. Burgio, Y. Zhang, C. Turnbull, X. Meng, P. Wu, E. Cho, L. A. Miosge, T. D. Andrews, M. A. Field, D. Tvorogov, A. F. Lopez, J. J. Babon, C. A. López, Á. Gónzalez-Murillo, D. C. Garulo, V. Pascual, T. Levy, E. J. Mallack, D. G. Calame, T. Lotze, J. R. Lupski, H. Ding, T. R. Ullah, G. D. Walters, M. E. Koina, M. C. Cook, N. Shen, C. de Lucas Collantes, B. Corry, M. P. Gantier, V. Athanasopoulos, C. G. Vinuesa, TLR7 gain-of-function genetic variation causes human lupus. *Nature* **605**, 349–356 (2022).
22. W. Song, O. Q. Antao, E. Condiff, G. M. Sanchez, I. Chernova, K. Zembrzuski, H. Steach, K. Rubtsova, D. Angeletti, A. Lemenze, B. J. Laidlaw, J. Craft, J. S. Weinstein, Development of Tbet- and CD11c-expressing B cells in a viral infection requires T follicular helper cells outside of germinal centers. *Immunity* **55**, 290–307.e5 (2022).

23. C. Wu, Q. Fu, Q. Guo, S. Chen, S. Goswami, S. Sun, T. Li, X. Cao, F. Chu, Z. Chen, M. Liu, Y. Liu, T. Fu, P. Hao, Y. Hao, N. Shen, C. Bao, X. Zhang, Lupus-associated atypical memory B cells are mTORC1-hyperactivated and functionally dysregulated. *Ann. Rheum. Dis.* **78**, 1090–1100 (2019).

24. L. M. Russell Knode, M. S. Naradikian, A. Myles, J. L. Scholz, Y. Hao, D. Liu, M. L. Ford, J. W. Tobias, M. P. Cancro, P. J. Gearhart, Age-Associated B Cells Express a Diverse Repertoire of $V_H$ and Vκ Genes with Somatic Hypermutation. *J. Immunol.* **198**, 1921–1927 (2017).

25. B. E. Barnett, R. P. Staupe, P. M. Odorizzi, O. Palko, V. T. Tomov, A. E. Mahan, B. Gunn, D. Chen, M. A. Paley, G. Alter, S. L. Reiner, G. M. Lauer, J. R. Teijaro, E. J. Wherry, Cutting Edge: B Cell-Intrinsic T-bet Expression Is Required To Control Chronic Viral Infection. *J. Immunol.* **197**, 1017–1022 (2016).

26. X. Han, S. Gu, S.-M. Hong, Y. Jiang, J. Zhang, C. Yao, Z. Yin, Z. Ye, H. Ding, S. Chen, D. Dai, N. Shen, Amelioration of Autoimmunity in a Lupus Mouse Model by Modulation of T-Bet-Promoted Energy Metabolism in Pathogenic Age/Autoimmune-Associated B Cells. *Arthritis Rheumatol.* **75**, 1203–1215 (2023).

27. P. Brescia, C. Schneider, A. B. Holmes, Q. Shen, S. Hussein, L. Pasqualucci, K. Basso, R. Dalla-Favera, MEF2B Instructs Germinal Center Development and Acts as an Oncogene in B Cell Lymphomagenesis. *Cancer Cell* **34**, 453–465.e9 (2018).

28. Y. Zhang, H. Wang, Integrin signalling and function in immune cells. *Immunology* **135**, 268–275 (2012).

29. J. L. Johnson, R. L. Rosenthal, J. J. Knox, A. Myles, M. S. Naradikian, J. Madej, M. Kostiv, A. M. Rosenfeld, W. Meng, S. R. Christensen, S. E. Hensley, J. Yewdell, D. H. Canaday, J. Zhu, A. B. McDermott, Y. Dori, M. Itkin, E. J. Wherry, N. Pardi, D. Weissman, A. Naji, E. T. L. Prak, M. R. Betts, M. P. Cancro, The Transcription Factor T-bet Resolves Memory B Cell Subsets with Distinct Tissue Distributions and Antibody Specificities in Mice and Humans. *Immunity* **52**, 842–855.e6 (2020).

30. J. J. O'Shea, A. Kontzias, K. Yamaoka, Y. Tanaka, A. Laurence, Janus kinase inhibitors in autoimmune diseases. *Ann. Rheum. Dis.* **72**, ii111–ii115 (2013).

31. P. G. Traves, B. Murray, F. Campigotto, R. Galien, A. Meng, J. A. Di Paolo, JAK selectivity and the implications for clinical inhibition of pharmacodynamic cytokine signalling by filgotinib, upadacitinib, tofacitinib and baricitinib. *Ann. Rheum. Dis.* **80**, 865–875 (2021).

32. M. J. van Helden, S. Goossens, C. Daussy, A.-L. Mathieu, F. Faure, A. Marçais, N. Vandamme, N. Farla, K. Mayol, S. Viel, S. Degouve, E. Debien, E. Seuntjens, A. Conidi, J. Chaix, P. Mangeot, S. de Bernard, L. Buffat, J. J. Haigh, D. Huylebroeck, B. N. Lambrecht, G. Berx, T. Walzer, Terminal NK cell maturation is controlled by concerted actions of T-bet and Zeb2 and is essential for melanoma rejection. *J. Exp. Med.* **212**, 2015–2025 (2015).

33. C. X. Dominguez, R. A. Amezquita, T. Guan, H. D. Marshall, N. S. Joshi, S. H. Kleinstein, S. M. Kaech, The transcription factors ZEB2 and T-bet cooperate to program cytotoxic T cell terminal differentiation in response to LCMV viral infection. *J. Exp. Med.* **212**, 2041–2056 (2015).

34. J. Klarquist, E. M. Janssen, The bm12 Inducible Model of Systemic Lupus Erythematosus (SLE) in C57BL/6 Mice. *J. Vis. Exp.* **105**, e53319 (2015).

35. M. Yokogawa, M. Takaishi, K. Nakajima, R. Kamijima, C. Fujimoto, S. Kataoka, Y. Terada, S. Sano, Epicutaneous application of toll-like receptor 7 agonists leads to systemic autoimmunity in wild-type mice: A new model of systemic Lupus erythematosus. *Arthritis Rheumatol.* **66**, 694–706 (2014).

36. A. Cossarizza, H.-D. Chang, A. Radbruch, A. Acs, D. Adam, S. Adam-Klages, W. W. Agace, N. Aghaeepour, M. Akdis, M. Allez, L. N. Almeida, G. Alvisi, G. Anderson, I. Andrä, F. Annunziato, A. Anselmo, P. Bacher, C. T. Baldari, S. Bari, V. Barnaba, J. Barros-Martins, L. Battistini, W. Bauer, S. Baumgart, N. Baumgarth, D. Baumjohann, B. Baying, M. Bebawy, B. Becher, W. Beisker, V. Benes, R. Beyaert, A. Blanco, D. A. Boardman, C. Bogdan, J. G. Borger, G. Borsellino, P. E. Boulais, J. A. Bradford, D. Brenner, R. R. Brinkman, A. E. S. Brooks, D. H. Busch, M. Büscher, T. P. Bushnell, F. Calzetti, G. Cameron, I. Cammarata, X. Cao, S. L. Cardell, S. Casola, M. A. Cassatella, A. Cavani, A. Celada, L. Chatenoud, P. K. Chattopadhyay, S. Chow, E. Christakou, L. Čičin-Šain, M. Clerici, F. S. Colombo, L. Cook, A. Cooke, A. M. Cooper, A. J. Corbett, A. Cosma, L. Cosmi, P. G. Coulie, A. Cumano, L. Cvetkovic, V. D. Dang, C. Dang-Heine, M. S. Davey, D. Davies, S. De Biasi, G. Del Zotto, G. V. Dela Cruz, M. Delacher, S. Della Bella, P. Dellabona, G. Deniz, M. Dessing, J. P. Di Santo, A. Diefenbach, F. Dieli, A. Dolf, T. Dörner, R. J. Dress, D. Dudziak, M. Dustin, C.-A. Dutertre, F. Ebner, S. B. G. Eckle, M. Edinger, P. Eede, G. R. A. Ehrhardt, M. Eich, P. Engel, B. Engelhardt, A. Erdei, C. Esser, B. Everts, M. Evrard, C. S. Falk, T. A. Fehniger, M. Felipo-Benavent, H. Ferry, M. Feuerer, A. Filby, K. Filkor, S. Fillatreau, M. Follo, I. Förster, J. Foster, G. A. Foulds, B. Frehse, P. S. Frenette, S. Frischbutter, W. Fritzsche, D. W. Galbraith, A. Gangaev, N. Garbi, B. Gaudilliere, R. T. Gazzinelli, J. Geginat, W. Gerner, N. A. Gherardin, K. Ghoreschi, L. Gibellini, F. Ginhoux, K. Goda, D. I. Godfrey, C. Goettlinger, J. M. González-Navajas, C. S. Goodyear, A. Gori, J. L. Grogan, D. Grummitt, A. Grützkau, C. Haftmann, J. Hahn, H. Hammad, G. Hämmerling, L. Hansmann, G. Hansson, C. M. Harpur, S. Hartmann, A. Hauser, A. E. Hauser, D. L. Haviland, D. Hedley, D. C. Hernández, G. Herrera, M. Herrmann, C. Hess, T. Höfer, P. Hoffmann, K. Hogquist, T. Holland, T. Höllt, R. Holmdahl, P. Hombrink, J. P. Houston, B. F. Hoyer, B. Huang, F.-P. Huang, J. E. Huber, J. Huehn, M. Hundemer, C. A. Hunter, W. Y. K. Hwang, A. Iannone, F. Ingelfinger, S. M. Ivison, H.-M. Jäck, P. K. Jani, B. Jávega, S. Jonjic, T. Kaiser, T. Kalina, T. Kamradt, S. H. E. Kaufmann, B. Keller, S. L. C. Ketelaars, A. Khalilnezhad, S. Khan, J. Kisielow, P. Klenerman, J. Knopf, H.-F. Koay, K. Kobow, J. K. Kolls, W. T. Kong, M. Kopf, T. Korn, K. Kriegsmann, H. Kristyanto, T. Kroneis, A. Krueger, J. Kühne, C. Kukat, D. Kunkel, H. Kunze-Schumacher, T. Kurosaki, C. Kurts, P. Kvistborg, I. Kwok, J. Landry, O. Lantz, P. Lanuti, F. LaRosa, A. Lehuen, S. LeibundGut-Landmann, M. D. Leipold, L. Y. T. Leung, M. K. Levings, A. C. Lino, F. Liotta, V. Litwin, Y. Liu, H.-G. Ljunggren, M. Lohoff, G. Lombardi, L. Lopez, M. López-Botet, A. E. Lovett-Racke, E. Lubberts, H. Luche, B. Ludewig, E. Lugli, S. Lunemann, H. T. Maecker, L. Maggi, O. Maguire, F. Mair, K. H. Mair, A. Mantovani, R. A. Manz, A. J. Marshall, A. Martínez-Romero, G. Martrus, I. Marventano, W. Maslinski, G. Matarese, A. V. Mattioli, C. Maueröder, A. Mazzoni, J. McCluskey, M. McGrath, H. M. McGuire, I. B. McInnes, H. E. Mei, F. Melchers, S. Melzer, D. Mielenz, S. D. Miller, K. H. G. Mills, H. Minderman, J. Mjösberg, J. Moore, B. Moran, L. Moretta, T. R. Mosmann, S. Müller, G. Multhoff, L. E. Muñoz, C. Münz, T. Nakayama, M. Nasi, K.

Neumann, L. G. Ng, A. Niedobitek, S. Nourshargh, G. Núñez, J.-E. O'Connor, A. Ochel, A. Oja, D. Ordonez, A. Orfao, E. Orlowski-Oliver, W. Ouyang, A. Oxenius, R. Palankar, I. Panse, K. Pattanapanyasat, M. Paulsen, D. Pavlinic, L. Penter, P. Peterson, C. Peth, J. Petriz, F. Piancone, W. F. Pickl, S. Piconese, M. Pinti, A. G. Pockley, M. J. Podolska, Z. Poon, K. Pracht, I. Prinz, C. E. M. Pucillo, S. A. Quataert, L. Quatrini, K. M. Quinn, H. Radbruch, T. R. D. J. Radstake, S. Rahmig, H.-P. Rahn, B. Rajwa, G. Ravichandran, Y. Raz, J. A. Rebhahn, D. Recktenwald, D. Reimer, C. Reis e Sousa, E. B. M. Remmerswaal, L. Richter, L. G. Rico, A. Riddell, A. M. Rieger, J. P. Robinson, C. Romagnani, A. Rubartelli, J. Ruland, A. Saalmüller, Y. Saeys, T. Saito, S. Sakaguchi, F. Sala-de-Oyanguren, Y. Samstag, S. Sanderson, I. Sandrock, A. Santoni, R. B. Sanz, M. Saresella, C. Sautes-Fridman, B. Sawitzki, L. Schadt, A. Scheffold, H. U. Scherer, M. Schiemann, F. A. Schildberg, E. Schimisky, A. Schlitzer, J. Schlosser, S. Schmid, S. Schmitt, K. Schober, D. Schraivogel, W. Schuh, T. Schüler, R. Schulte, A. R. Schulz, S. R. Schulz, C. Scottá, D. Scott-Algara, D. P. Sester, T. V. Shankey, B. Silva-Santos, A. K. Simon, K. M. Sitnik, S. Sozzani, D. E. Speiser, J. Spidlen, A. Stahlberg, A. M. Stall, N. Stanley, R. Stark, C. Stehle, T. Steinmetz, H. Stockinger, Y. Takahama, K. Takeda, L. Tan, A. Tárnok, G. Tiegs, G. Toldi, J. Tornack, E. Traggiai, M. Trebak, T. I. M. Tree, J. Trotter, J. Trowsdale, M. Tsoumakidou, H. Ulrich, S. Urbanczyk, W. van de Veen, M. van den Broek, E. van der Pol, S. Van Gassen, G. Van Isterdael, R. A. W. van Lier, M. Veldhoen, S. Vento-Asturias, P. Vieira, D. Voehringer, H.-D. Volk, A. von Borstel, K. von Volkmann, A. Waisman, R. V. Walker, P. K. Wallace, S. A. Wang, X. M. Wang, M. D. Ward, K. A. Ward-Hartstonge, K. Warnatz, G. Warnes, S. Warth, C. Waskow, J. V. Watson, C. Watzl, L. Wegener, T. Weisenburger, A. Wiedemann, J. Wienands, A. Wilharm, R. J. Wilkinson, G. Willimsky, J. B. Wing, R. Winkelmann, T. H. Winkler, O. F. Wirz, A. Wong, P. Wurst, J. H. M. Yang, J. Yang, M. Yazdanbakhsh, L. Yu, A. Yue, H. Zhang, Y. Zhao, S. M. Ziegler, C. Zielinski, J. Zimmermann, A. Zychlinsky, Guidelines for the use of flow cytometry and cell sorting in immunological studies (second edition). *Eur. J. Immunol.* **49**, 1457–1973 (2019).

37. A. P. Heinen, F. Wanke, S. Moos, S. Attig, H. Luche, P. P. Pal, N. Budisa, H. J. Fehling, A. Waisman, F. C. Kurschus, Improved method to retain cytosolic reporter protein fluorescence while staining for nuclear proteins. *Cytometry A* **85**, 621–627 (2014).

38. T. G. Montague, J. M. Cruz, J. A. Gagnon, G. M. Church, E. Valen, CHOPCHOP: A CRISPR/Cas9 and TALEN web tool for genome editing. *Nucleic Acids Res.* **42**, W401–W407 (2014).

39. D. Conant, T. Hsiau, N. Rossi, J. Oki, T. Maures, K. Waite, J. Yang, S. Joshi, R. Kelso, K. Holden, B. L. Enzmann, R. Stoner, Inference of CRISPR Edits from Sanger Trace Data. *CRISPR J.* **5**, 123–130 (2022).

40. Y. Meng, J. Ma, C. Yao, Z. Ye, H. Ding, C. Liu, J. Li, G. Li, Y. He, J. Li, Z. Yin, L. Wu, H. Zhou, N. Shen, The NCF1 variant p.R90H aggravates autoimmunity by facilitating the activation of plasmacytoid dendritic cells. *J. Clin. Invest.* **132**, e153619 (2022).

41. G. X. Y. Zheng, J. M. Terry, P. Belgrader, P. Ryvkin, Z. W. Bent, R. Wilson, S. B. Ziraldo, T. D. Wheeler, G. P. McDermott, J. Zhu, M. T. Gregory, J. Shuga, L. Montesclaros, J. G. Underwood, D. A. Masquelier, S. Y. Nishimura, M. Schnall-Levin, P. W. Wyatt, C. M. Hindson, R. Bharadwaj, A. Wong, K. D. Ness, L. W. Beppu, H. J. Deeg, C. McFarland, K. R. Loeb, W. J. Valente, N. G. Ericson, E. A. Stevens, J. P. Radich, T. S. Mikkelsen, B.

J. Hindson, J. H. Bielas, Massively parallel digital transcriptional profiling of single cells. *Nat. Commun.* **8**, 14049 (2017).

42. T. Stuart, A. Butler, P. Hoffman, C. Hafemeister, E. Papalexi, W. M. Mauck 3rd, Y. Hao, M. Stoeckius, P. Smibert, R. Satija, Comprehensive Integration of Single-Cell Data. *Cell* **177**, 1888–1902.e21 (2019).
43. D. Kim, B. Langmead, S. L. Salzberg, HISAT: A fast spliced aligner with low memory requirements. *Nat. Methods* **12**, 357–360 (2015).
44. S. Anders, P. T. Pyl, W. Huber, HTSeq—A Python framework to work with high-throughput sequencing data. *Bioinformatics* **31**, 166–169 (2015).
45. M. I. Love, W. Huber, S. Anders, Moderated estimation of fold change and dispersion for RNA-seq data with DESeq2. *Genome Biol.* **15**, 550 (2014).
46. B. Langmead, S. L. Salzberg, Fast gapped-read alignment with Bowtie 2. *Nat. Methods* **9**, 357–359 (2012).
47. A. Tarasov, A. J. Vilella, E. Cuppen, I. J. Nijman, P. Prins, Sambamba: Fast processing of NGS alignment formats. *Bioinformatics* **31**, 2032–2034 (2015).
48. A. R. Quinlan, I. M. Hall, BEDTools: A flexible suite of utilities for comparing genomic features. *Bioinformatics* **26**, 841–842 (2010).
49. F. Ramírez, D. P. Ryan, B. Grüning, V. Bhardwaj, F. Kilpert, A. S. Richter, S. Heyne, F. Dündar, T. Manke, deepTools2: A next generation web server for deep-sequencing data analysis. *Nucleic Acids Res.* **44**, W160–W165 (2016).
50. Y. Zhang, T. Liu, C. A. Meyer, J. Eeckhoute, D. S. Johnson, B. E. Bernstein, C. Nusbaum, R. M. Myers, M. Brown, W. Li, X. S. Liu, Model-based analysis of ChIP-Seq (MACS). *Genome Biol.* **9**, R137 (2008).
51. C. E. Grant, T. L. Bailey, W. S. Noble, FIMO: Scanning for occurrences of a given motif. *Bioinformatics* **27**, 1017–1018 (2011).
